# In Silico Genome-Scale Analysis of Molecular Mechanisms Contributing to the Development of a Persistent Infection with Methicillin-Resistant *Staphylococcus aureus* (MRSA) ST239

**DOI:** 10.3390/ijms232416086

**Published:** 2022-12-16

**Authors:** Olga Dmitrenko, Andrey Chaplin, Anna Balbutskaya, Tamara Pkhakadze, Sergey Alkhovsky

**Affiliations:** 1 N.F. Gamaleya National Research Center for Epidemiology and Microbiology, Ministry of Health of Russian Federation, Gamaleya Street 18, 123098 Moscow, Russia; 2 Department of Microbiology and Virology, Pirogov Russian National Research Medical University, 117997 Moscow, Russia; 3 Faculty of Animal Engineering and Biotechnology, Department of Large Animal Husbandry, Saint-Petersburg State Agrarian University, Peterburgskoye Shosse, House 2, Pushkin, 196601 Saint-Petersburg, Russia; 4 Federal State Budgetary Institution, National Medical Research Center for Traumatology and Orthopedics Named after N.N. Priorov, 127299 Moscow, Russia; 5 D.I. Ivanovsky Institute of Virology, National Research Center for Epidemiology and Microbiology, Ministry of Health of Russian Federation, Gamaleya Street 18, 123098 Moscow, Russia

**Keywords:** *Staphylococcus aureus*, MRSA_ST239_, osteomyelitis, genome, features, pathogenesis, adaptation, chronic, infection

## Abstract

The increasing frequency of isolation of methicillin-resistant *Staphylococcus aureus* (MRSA) limits the chances for the effective antibacterial therapy of staphylococcal diseases and results in the development of persistent infection such as bacteremia and osteomyelitis. The aim of this study was to identify features of the MRSA_ST239_ 0943-1505-2016 (SA943) genome that contribute to the formation of both acute and chronic musculoskeletal infections. The analysis was performed using comparative genomics data of the dominant epidemic *S. aureus* lineages, namely ST1, ST8, ST30, ST36, and ST239. The SA943 genome encodes proteins that provide resistance to the host’s immune system, suppress immunological memory, and form biofilms. The molecular mechanisms of adaptation responsible for the development of persistent infection were as follows: amino acid substitution in PBP2 and PBP2a, providing resistance to ceftaroline; loss of a large part of prophage DNA and restoration of the nucleotide sequence of beta-hemolysin, that greatly facilitates the escape of phagocytosed bacteria from the phagosome and formation of biofilms; dysfunction of the AgrA system due to the presence of *psm-mec* and several amino acid substitutions in the AgrC; partial deletion of the nucleotide sequence in genomic island vSAβ resulting in the loss of two proteases of Spl—operon; and deletion of SD repeats in the SdrE amino acid sequence.

## 1. Introduction

*Staphylococcus aureus* is a pathogenic microorganism that can cause both acute and chronic human diseases. Recurrent bacteremia and osteomyelitis are the most severe forms of the chronic infectious process caused by this microorganism, that are very difficult to treat using traditional methods [1,2,3]. Osteomyelitis is an infectious process that occurs in bone tissue and leads to its destruction. The penetration of the pathogen into the bone tissue and the further development of the inflammatory process can be carried out both by the hematogenous route and as a result of trauma or surgical intervention. When the pathogen reaches the bone surface, it causes a strong inflammatory response, followed by destruction of bone tissue and loss of its vascularization. As a result, individual parts of the bone die off and are separated from healthy bone, forming bone sequesters. The area of dead tissue is inaccessible to immune cells or antibiotics, leading to the development of a persistent infection [4,5,6]. Under physiological conditions, bone is constantly undergoing remodeling, a complex process that includes both bone formation and bone resorption. These processes are finely controlled by the combined action of osteoblasts and osteoclasts, which are sequentially responsible for the synthesis of new bone and the resorption of old bone. Bone formation follows a cascade of complex events that include the proliferation of primitive stem cells, their differentiation into osteoblasts, matrix formation, and final mineralization. Bone resorption is carried out by activated multinuclear osteoclasts, which have differentiated from mononuclear cells—precursors of the monocyte–macrophage lineage of the bone marrow. The dominant cytokine that regulates differentiation and proliferation of osteoclasts is RANKL (receptor activator of nuclear factor–kB ligand). It is produced by osteoclasts in membrane-bound and soluble forms [4,7,8].

In recent decades, a considerable amount of experimental evidence has emerged indicating that certain pathogenicity factors of *S. aureus* play a key role in the development of osteomyelitis. It has been found that *S. aureus* strains, which can produce protein products capable of interacting with bone extracellular matrix proteins (sialoprotein, collagen, and fibronectin), are associated with the development of osteomyelitis and arthritis [7,9,10]. It has been shown under in vitro that *S. aureus* protein A is able to bind to osteoblasts through TNFα receptors, causing caspase 3 activation and formation of gaps in the cell membrane, leading to inhibition of osteoblast proliferation and then to their death. *S. aureus* also induces RANKL expression by osteoblasts and enhances osteoclastogenesis and osteoclast proliferation [11,12]. Increased resorptive activity of osteoclasts leads to further weakening of the bone tissue. During the *S. aureus* infection, the processes of deposition of phosphates and calcium in the bone tissue are disrupted, and mineralization is inhibited [12,13,14].

Difficulties in the treatment of diseases such as osteomyelitis are complicated by the prevalence of multiple antibiotic-resistant methicillin-resistant *S. aureus* (MRSA). Numerous epidemiological studies, as well as the results of whole genome sequencing, indicate that only a few genetic linages are predominant, both among hospital (HA-MRSA) and community-acquired MRSA (CA-MRSA). According to multilocus sequencing (MLST) data, the most common pathogens of hospital infections are representatives of clades or clonal complexes (CC) CC5, CC8 (including CC8/CC239), CC22, CC30, and CC45 [15,16,17,18]. An analysis of publications over the past 20 years has shown that the vast majority of MRSA strains isolated from patients with osteomyelitis also belong to these clades [19]. 

There is now no doubt that most *S. aureus* infections result from the combined action of various microbial products. However, the contribution of particular pathogenicity factors of *S. aureus* to virulence in humans has not yet been studied enough. Most studies are aimed at studying the pathogenetic role of numerous *S. aureus* toxins [20,21,22,23,24]. Their role in the development of various acute forms of infection, such as sepsis, toxic shock, endocarditis, pneumonia, and food poisoning, has been proven [22,25,26,27,28]. However, it is becoming clear that the main pathogenetic mechanisms that allow *S. aureus* to develop persistent forms of infection are the ability to reprogram the action of the macroorganism’s immune system, cell invasion resulting in eukaryotic cell death, and biofilm formation [29,30,31,32]. Significant progress in understanding osteomyelitis pathogenesis can be introduced by studies of comparative genomics of bacterial isolates obtained from infection foci of the osteomuscular system. However, such investigations are still extremely few [33,34].

The purpose of the work: to perform whole genome sequencing and to conduct genomic analysis of the representative of the epidemic clone MRSA ST239, isolated from patients with chronic musculoskeletal infection, in order to evaluate the genomic features that contributed to the development of a persistent form of infection.

For comparison, fully annotated genomes of *S. aureus* strains of several sequence types, both hospital and community-acquired, isolated for more than 60 years, were selected from the NCBI GenBank (in Materials and Methods). The results of genomic analysis revealed that one of the main strategies developed by *S. aureus*, and which is clearly reflected in its genomes, is the multiple duplication of proteins that perform key virulent functions. A unique feature of the pathogen is its ability to sacrifice a part of the mobile genetic pool in order to more effectively tune the work of the virulence gene repertoire and acquire additional advantages in solving urgent problems of confrontation with the host. For fine regulation of virulence, cooperation of the work of numerous pathogenicity factors at various stages of infection allows the pathogen to successfully maintain the inflammatory process in various environments and host tissues. It is becoming increasingly clear that strategies for both the prevention and treatment of staphylococcal infections must be simultaneously targeted at multiple molecular targets of the pathogen, and the drugs created should be adapted to different types of infectious process.

## 2. Results

### 2.1. Assembly Characteristics and Structural Features of the Genome

According to the whole genome sequencing results, the total length of the reads was 1,919,469,985 nucleotides, which indicated more than 250-fold genome coverage. According to the assembly results, 118 contigs were formed with a total length of 2,895,449 bp with a G + C content of 32.7%, which corresponds to the studied species. A total of 3124 genes have been identified, including: 2936 protein-coding genes, 80—ribosomal RNA, 60—transfer RNA, and 4—non-coding RNA. Several types of transposable genetic elements have been identified, including SCC*mec* III 1.1.4, genomic islands υSAα, υSAβ, and transposon Tn6072. In addition, fragments of the SaPI1 pathogenicity island were identified, one of which, about 3000 bp long, carried the genes encoding enterotoxins Q and K, as well as 5 incomplete prophage regions with a length: 28; 25.8; 19.8; 9.7; and 8.3 bp. No plasmids were found, according to PlasmidFinder. Based on a number of genetic markers (absence of the gene encoding fibrinogen-binding protein B, absence of the *mer* operon in SCC*mec,* absence of the *splE* in the serine protease operon, and a number of other features), the genome was assigned to the Eurasian subclone ST239 according to the classification [35]. The results of whole genome sequencing of *S. aureus* 943 are presented in DDBJ/ENA/GenBank under number QLNS00000000, as well as in the SRA NCBI read archive (accession PRJNA476233). The features of genome organization were considered in more detail earlier [36].

### 2.2. Genotyping and Sensitivity to Antibiotics

The results of multilocus sequencing performed in silico confirmed that the strain belongs to the epidemic clone MRSA ST239. When analyzing the results of spa typing, a unique sequence of short nucleotide repeats (SSR) was found: 15-12-16-02-153-24, which was entered into the spa server database as a new spa type t-18470. It differs from the widespread t-030 (15-12-16-02-24-24) by the presence of one nucleotide substitution (C-T) in the structure of the fifth SSR, which led to the replacement of the fifth repeat r24 by r153. This substitution was not accompanied by amino acid substitution. SA943 had multiple antibiotic resistance including beta-lactams (ampicillin, amoxicillin clavulanate, oxacillin, cephalosporins of I-III generations) tetracycline, chloramphenicol, and aminoglycosides (gentamicin, tobramycin) and showed an inducible type of resistance to lincosamides and macrolides. Resistance to fluoroquinolones (ciprofloxacin and levofloxacin) was due to amino acid substitutions in proteins: NorA, DNA gyrase subunits (GyrA, GyrB), and topoisomerase IV DNA subunits (GrlA and GrlB). Resistance to rifampicin was the result of mutations in the rpoB gene. A specific feature of the strain was resistance to ceftaroline (MIC 4 mg/L), a fifth-generation cephalosporin developed for the treatment of infections caused by MRSA, as a result of amino acid substitutions in proteins PBP2 (R262C; V627G) and PBP-2a (N146K; E246G). SA943 remained sensitive to vancomycin (MIC = 1 µg/mL), daptomycin (MIC = 1 µg/mL), linezolid (MIC = 2 µg/mL), tigecycline (MIC ˂ 0.25 µg/mL), fusidic acid MIC ≤ 0.5 µg/mL), and trimethoprim/sulfameioxazole ˂ 0.5/9.5 µg/mL.

### 2.3. Virulome Features

Sequencing of the genomes of *S. aureus* isolates, isolated from humans and animals, showed that most of the genes encoding pathogenicity factors are present in the core part of the genome (Supplementary Table S1). However, a significant part is located on mobile genetic elements and, therefore, is not present in all strains of the pathogen [37,38,39]. Moreover, even conservative genes exhibit sequence polymorphism, which can affect both virulence and limit the possibility of creating effective vaccines against this pathogen. In this regard, it seemed appropriate not only to characterize the set of pathogenicity genes present in the strain but also to compare the amino acid composition of some of them with similar ones in other strains.

#### 2.3.1. MSCRAMM Family Proteins That Are the Key Molecules Causing Inflammation in the Bone Tissue

Adhesion and subsequent colonization of host tissues are critical steps in the pathogenesis of *S. aureus* infections. In these processes, the leading role belongs to adhesins—proteins covalently bound to the cell wall of a microorganism, representatives of the MSCRAMM family (microbial proteins that recognize cell adhesion molecules) [32,40,41]. All MSCRAMMs have a similar structural organization and are involved in the binding of several specific host ligands, which determines their different role in the pathogenesis of the infectious process [42]. Genes encoding aggregation factors (ClfA, ClfB), fibronectin binding protein (FnbA), extracellular fibrinogen binding protein (Efb), extracellular adhesion proteins Emp, Eap/map, collagen binding protein (Cna), surface protein SasG, as well as proteins of the Sdr family included in this group, containing the serine-aspartate dipeptide repeats SdrC, SdrD, SdrE, have been identified in the studied genome.

##### Protein A Structure Features

**Figure 1:**
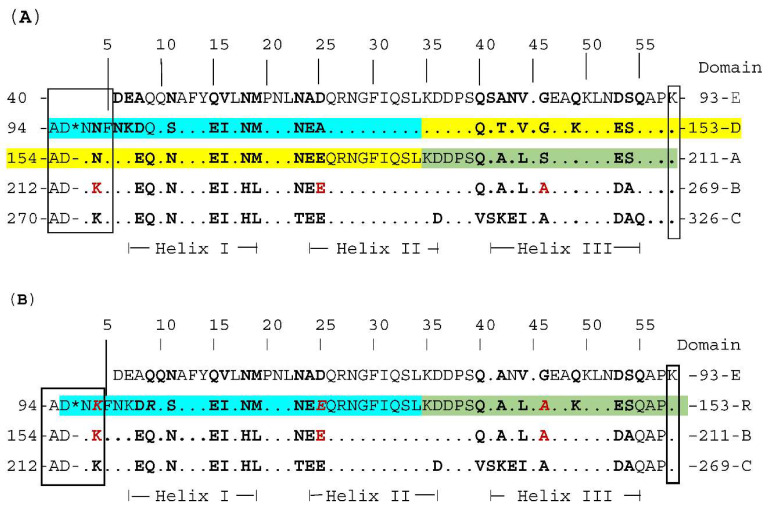
Structural features of protein A (SpA) domains (sequence aliment protein A binding domains) in *S. aureus* NCTC8325 (**A**) and *S. aureus* SA943 (**B**). Note: Letters designate structural domains according to Jo. A. Capp et al. (2014) [46]. Letter R denotes the formed recombinant domain. Dots indicate identical amino acids; domain areas differing in amino acid sequences are marked in bold type. The linker region between domains is boxed in black. Each of the domains consists of 3 helix bundles connected by short linkers; * Amino acid insertion (AQQ) between domains E and D (**A**) or E and R (**B**). The part of the D domain involved in the formation of R domain is highlighted in blue; the part of the A domain involved in the formation of the R domain is highlighted in green; the deletion region of D and A domains is shown in yellow. The original amino acid substitution in the R domain is highlighted in black italics; red italics highlight amino acids in the R domain that are specific to domain B and C, but representing substitutions in relation to domains D and A; Amino acids that are specific for the B domain and absent in the fragments of the D and A domains that form the R domain are highlighted in red normal fonts.

According to current data, in the processes of attachment and internalization into osteoblasts, the key role belongs to such representatives of MSCRAMM as protein A and fibronectin-binding proteins [9,13]. Protein A is a 42 kDa protein covalently linked to the cell wall via a carboxyl end. In the reference NCTC8325 genome, the protein consists of five repetitive similar domains (E, D, A, B, C) that are attached to the cell wall surface via the Xr region, which is represented by a variable number of short repeats, usually consisting of eight amino acids. Variability in the number and nucleotide sequence of repeating elements in this region is used as an epidemiological marker, but the biological properties of this region have not yet been studied. An analysis of the crystal structure showed that the SpA domains have the form of three-helix bundles, in which the helices are connected by short (6–9 amino acid residues) movable linkers. Each domain is folded independently. Each SpA domain can bind with high affinity to the Fc region of immunoglobulin G and the Fab region of immunoglobulin VH3 subclasses (IgG, IgA, IgM, and IgE). Analysis of the features of the interaction of protein A with immunoglobulins revealed that on one side, the amino acid residues of helices I and II bind to the Fc region of IgG, while the residues from helices II and III on the other side bind IgM. Residues from helix II that bind Fc differ from those that bind Fab, with the exception of one glutamine (Gln32 in SpA domain D) [43,44,45,46]. The interaction of SpA with IgG leads to disruption in the process of recognition, opsonization, and subsequent phagocytosis of bacteria by neutrophils. Protein binding to IgM triggers cross-linking of B-cell receptors, proliferative expansion, and apoptotic collapse of activated B-cells [47,48]. Protein A can also bind directly to osteoblast progenitors via the tumor necrosis factor receptor (TNFR-1), without the presence of extracellular matrix components. When analyzing amino acid substitutions in the D domain of the protein under experimental conditions, it was found that the same amino acid residues that interact with the Fc region of IgG are involved in the binding and activation of TNFR-1. Interaction with TNFR-1 leads to the generation of a signal that causes osteoblast apoptosis, preventing the formation of new bone and its mineralization processes. Protein A can increase the release soluble form of TNFR-1, resulting in neutralization of circulating TNFα and attenuation of the host’s inflammatory response. In addition, SpA enhances osteoclast proliferation and enhances their resorptive activity through induction of RANKL expression by osteoblasts [13,48,49]. The results of the analysis of the amino acid composition of protein A in the analyzed strains are presented in Supplementary Table S2a,b. Significant variability in the number of amino acids in the sequence of protein A was revealed, not only in strains of different sequence types, but also among representatives of ST239. When compared with protein A in strain NCTC8325, the identity of the amino acid composition ranged from 96.56 to 75.57% at 100% coverage. The differences were due to the presence, as a rule, of several deletions affecting both the immunoglobulin-binding region and the C-terminus. At the same time, in all strains of ST239, a deletion of 58 amino acid residues in the immunoglobulin-binding region (130–187 aa) was detected, which led to the loss of most of the D domain, including helices II and III, as well as part of domain A, including helix I and partially helix II, including key amino acid residues required for interaction with Fab fragments of immunoglobulins. The aforementioned deletion resulted in the formation of a recombinant domain bearing the amino acid composition of the three domains D, A, and B and a reduction in the total number of immunoglobulin-binding domains from five to four. The QQ motif at the beginning of helix I of all domains underwent changes and became RQ in R domain, as a result of amino acid substitution. Strain SA943 had another large deletion of 48 aa at the C end (positions 360–407). A similar set of deletions was found only in strain T0131, isolated in China (Figure 1).

In addition, SA943 has a substitution of aspartic acid for lysine (D461A). A similar substitution was also present in strain To131. It should be noted that other ST239 strains (TW20, Z172, V521, JKD 6008, and Be62) have three deletions in the C region (Supplementary Table S2b).

##### Structure and Functions of Fibronectin-Binding Proteins

Fibronectin (Fn) binding proteins FnBPA and FnBPB are involved in the invasion of a number of non-phagocytic cell lines, bind to elastin, plasminogen, and histones (FnBPB), and play an important role in biofilm formation. Both proteins can bind to osteoblasts [42,50,51,52]. Interaction with these cells leads to the internalization of the microorganism into phagocytic vesicles of osteoblasts, which makes the microbe less vulnerable to the action of antibiotics and host immune defense factors. Since only the gene encoding FnbA was identified in the SA943 genome, the amino acid sequence of this particular protein was analyzed (Supplementary Table S3a,b). Several regions are distinguished in the protein structure. The N-terminus is represented by a signal sequence (S) followed by a variable fragment (Nv), a fibrinogen-binding A-domain consisting of three folded IgG-like subdomains (from N1 to N3) [52,53]. The combination of N2 and N3 subdomains forms a hydrophobic groove, which interacts with fibrinogen [42]. Domain A is followed by an unstructured fibronectin-binding region responsible for invasion processes and consisting of 11 repeats. The C-terminus contains proline-rich repeats (PRR), cell wall (W) and membrane (M) attachment domains, including the L-P-E-T-G signaling motif recognized by sortase A, and a short cytosolic region [40,54]. The N2 subdomain of FnBPA can bind to plasminogen. The uptake of plasminogen from serum and its conversion to plasmin by host tissue activators or staphylokinase promotes the degradation of opsonins and facilitates the spread of bacteria in infected tissues. The A domain also binds with high affinity to tropoelastin. The interaction occurs at several sites in repeating sections of the tropoelastin sequence [55,56]. It has been shown that only FnBPA is sufficient for the invasion of a eukaryotic cell [57]. Unstructured Fn binding repeats bind individually to the N-terminal domain of fibronectin and have significant sequence variations that affect the degree of ligand affinity of each repeat. One FnBPA molecule can interact with six to nine fibronectin molecules, which form a kind of bridge with α5β integrins on the surface of a eukaryotic cell and trigger the process of invasion. Upon binding to fibronectin, the Fn-binding repeat region acquires an ordered secondary structure [42,58]. FnBPA and FnBPB expressed by clinically relevant HA-MRSA strains are involved in biofilm formation [51,59,60].

The amino acid composition of FnBPA differed significantly in the studied genomes, resulting from both amino acid substitutions and deletions (Supplementary Table S3a,b). The protein size varied from 1042 in the 55/2053 strain to 741 amino acid residues in the Newman strain, while in the reference NCTC8325 genome it was 1018 amino acid residues, and in the SA943 strain it was 990 amino acid residues. SA943 had four amino acid substitutions in the repeat region (I936V; A970V; P975A; K993N). Identical substitutions were also found in strain T0131. The analyzed ST239 strains, such as the ST8 strains (NCTC 8325 and FPR3757), carried a 24 amino acid deletion affecting the end of the fibronectin-binding region and the repeat region. In addition, strains SA943 and T0131 had an additional deletion of 28 aa in the repeating region. Unlike *S. aureus* NCTC 8325, strain SA943, such as other analyzed strains of ST8 and ST239, had one amino acid substitution in the A domain and in the fibronectin-binding region. The largest number of amino acid substitutions was found in MW2, N315, MRSA252, and 55/2053 genomes, which were localized in all protein regions.

##### Characteristics of Sdr Family Proteins, Containing Serine-Aspartate Dipeptide Repeats: SdrC, SdrD, and SdrE

Another group of surface proteins of the MSCRAMM family, encoded by the *sdrC*, *sdrD*, and *sdrE* genes located in tandem in the Sdr locus, with a length of approximately 2.8, 3.9, and 3.5 kb, respectively. The Sdr proteins have a comparable structural organization. The signal peptide is followed by the A domain, which is similar in size among different members of the Sdr family, but only 20–30% of the amino acid residues are identical. The A domain is followed by 2, 3, or 5 additional sequences consisting of 110–113 residues (B motifs), which are repeated in tandem in SdrC, SdrE, and SdrD, respectively. The function of B-domains remains unknown. It is believed that the B domains may act as spacers or hinges, regulating the distance between the A ligand-binding region and the microbial cell surface, giving *S. aureus* the ability to flexibly interact with various host proteins. Behind it, there are segments containing different amounts of Ser-Asp dipeptides or SD repeats (R-region). The C-terminal end (M region) of Sdr proteins is involved in the attachment of proteins to the bacterial cell wall. The presence of structural differences indicates that Sdr proteins play different roles in the pathogenicity of *S. aureus* [61,62,63,64]. However, a ligand has been identified only for bone sialo-binding protein (Bbp), which is an allelic variant of SdrE [65]. Although SdrC is present in all genomes tested, SdrD appears to play a key role during colonization and infection. Expression of SdrD promotes the adhesion of *S. aureus* to keranocytes and enhances pathogen virulence during systemic infection. This protein is able to inhibit the native bacterial killing of *S. aureus* by neutrophils independently of other proteins of the microorganism and, thereby, contribute to the survival of the pathogen in whole blood [66]. SdrE is also involved in immune evasion processes and interacts with components of the complement system, C4b-binding protein (C4BP), the classical complement regulator, and the major human fluid-phase complement regulator factor H to prevent bacterial-mediated opsonization and killing [67,68,69]. Bearing in mind that a number of studies report a higher prevalence of the *sdrD* and *sdrE* among *S. aureus* strains responsible for bone infections [70,71], we analyzed the presence genes of the Sdr locus and the amino acid sequence of the encoded proteins. Bioinformatics analysis showed that all three proteins are encoded in the SA943 genome: SdrC, SdrE, and SdrD, while the SdrD protein turned out to be conserved (Supplementary Tables S2 and S4). Its length in SA943, as in the reference strain Col, was 1381 amino acid residues with 100% identity. In other ST239 strains, the identity of the amino acid sequence of the protein with that of the Col strain was 97–100%; the differences, as a rule, were due only to the number of SD repeats. At the same time, this protein turned out to be variable in the ST30 and ST5 strains; its identity with the analogous protein in the Col strain in *S. aureus* 55/2053 and *S. aureus* N315 ranged from 82.77 to 93.77% with coverage not exceeding 81%. The largest number of amino acid substitutions was found in the A domain. Analysis of the amino acid sequence of SdrE revealed the following. The number of amino acid residues ranged from 1166 in the reference strain Col to 1029 in strain SA943 (Supplementary Table S5). This variability was mainly due to differences in the number of SD repeats in the R region. Thus, the SA943 strain had a deletion of 68 SD repeats, the largest number among all analyzed genomes. Additionally, several strains of ST239, including SA943, had a 5 amino acid deletion (Thr-Ser-Glu-Pro-Ser), between 174 and 178 amino acid residues in the A domain. In addition, a substitution of lysine for asparagine (Lys227Asn) was found in the A domain, which was found in the genomes of strains of several sequence types. Curiously, all strains, except TW20, had an arginine to serine substitution localized in the B2 subdomain. It should be noted that the N2–N3 subdomain region of domain A, which is believed to be a ligand-binding region, was highly conserved.

##### Collagen-Binding Protein Cna

Another multifunctional representative of MSCRAMM is collagen-binding protein Cna. It mediates attachment to two structurally and functionally distinct host proteins, the complement system protein C1q, and the extracellular matrix protein laminin, using different mechanisms [72]. Cna contains an N-terminal signal peptide, a non-repetitive region A with domains N1, N2, and N3; tandem B repeats and a cell wall attachment region containing the LPxTG domain; a transmembrane segment, and a short positively charged cytoplasmic tail. Due to the interaction of C1q (having six collagen-like domains), Cna is able to inhibit the classical pathway of complement fixation [73,74]. The *cna* gene was detected only in the genomes of ST239. Protein (RAM46073.1) consists of 809 amino acid residues who showed 100% identity within the group (the data are not provided).

#### 2.3.2. Toxins and Peptides That Are Active Participants of Immune Evasion

##### Cytolytic Toxins

Various experimental models have shown that *S. aureus* has developed numerous strategies that allow it to both resist the native immune response and block the formation of a secondary immune response of the macroorganism and, thereby, contribute to the occurrence of infection relapses [29,30,75]. Among them, one of the key roles belongs to cytolytic toxins capable of killing host myeloid cells. In the described genome, cytolytic or membrane-damaging toxins were identified, including hemolysins: alpha-hemolysin (Hla), gamma-hemolysins (HlgAB, HlgCB), beta-hemolysin (Hlb), delta-hemolysin (Hld), hemolysin III and leukocidins: LukE/D and LukG/H. These proteins can cause the death of erythrocytes and myeloid cells (neutrophils, lymphocytes, dendritic cells) by binding to receptors on their surface. Under experimental conditions, it has been shown that alpha-hemolysin can promote the release of *S. aureus* from the neutrophil phagosome and contribute to the generalization of infection. Purified HlgAB and HlgCB cause lysis of neutrophils, monocytes, and macrophages. LukA/B (otherwise LukG/H) causes cytotoxic death of human neutrophils. LukE/D triggers the lysis of macrophages, dendritic cells, and T lymphocytes, including T-memory cells. Thus, this group of pathogenicity factors is able to suppress both primary and secondary immune responses [21,23,76,77]. Analysis of the amino acid sequence of the cytolytic toxin gene products revealed a high conservation of these proteins in SA943 (Supplementary Table S6). As in all representatives of ST239, the *hla* gene product differed from the canonical protein sequence in the NCTC 8325 strain by the amino acid substitution of arginine for threonine (R4T) in the protein signal sequence. Amino acid sequences of proteins LukE, LukD, subunits A, B, C of gamma hemolysin, and both subunits of leukocidin LukGH showed 100% homology with those sequences in *S. aureus* NCTC 8325. The original difference of SA943 was the substitution of isoleucine for threonine (Ile96Thr) in the amino acid sequence of hemolysin III. A feature of the SA943 genome was the presence of an intact beta-hemolysin gene (*hlb*) due to a partial deletion of the beta-converting prophage genome. The ϕSa3int family is the most common staphylococcal phage family, whose representatives are able to integrate into the chromosome into the *hlb* encoding β-toxin, inactivate it, and cause lysogenic conversion. ϕsa3int prophages can encode several types of immune escape cluster (IEC) genes, consisting of immunomodulators: staphylokinase (SAK), staphylococcal complement inhibitor (SCIN), staphylococcal enterotoxin A (SEA), and *S. aureus* chemotaxis-inhibiting protein (CHIPS). Depending on the presence/absence of genes encoding these additional proteins, integration results in single conversion (*hlb* interrupted), double conversion (*hlb* interrupted, SAK+), or triple conversion (*hlb* interrupted, SAK+, SEA+) events [78,79]. It has long been assumed that Hlb has little effect on the pathogenesis of diseases caused by *S. aureus.* However, it has recently been shown that by possessing at least two binding sites, which provide it with the activity by sphingomyelinase and DNA biofilm ligase activity, it promotes host colonization, modulates the immune response to infection, stimulates biofilm formation, and increases the severity of life-threatening diseases such as pneumonia and infective endocarditis [80,81,82,83,84].

##### Phenol Soluble Modulins (PSMs)

Another group of *S. aureus* cytolytic pathogenicity factors is represented by short peptides called phenol soluble modulins (PSMs). The PSM family consists of several peptides: PSM 1–4 α-type, PSM 1–2 β-type, δ-toxin, and PSM-*mec* encoded by only a few types of SCC*mec* [24,85]. PSMs can act both intracellularly and extracellularly and cause osteoblast death [86]. Due to their amphipathic structure, they are able to destroy the lipid layer of the eukaryotic cell membrane, causing its death. *S. aureus* microbial cells that invade nonprofessional phagocytes, such as osteoblasts, initially remain trapped in phagosomes. Accumulating in the limited space of the phagosome, PSM, together with other toxins, such as delta-toxin and beta-hemolysin, disrupt the permeability of its membrane, promoting the release of *S. aureus* into the cytosol [87,88]. However, the exact mechanism by which PSMs contribute to host cell death has not been fully elucidated yet. It was shown that, unlike other PSMs, PSMmec is involved in the regulation of the expression of pathogenicity factors. SCC*mec* srRNA suppresses translation of the Agr regulatory locus and, thus, attenuates MRSA virulence [89]. Analysis of the amino acid sequence of PSM gene products (Supplementary Table S7) showed their high conservatism in all analyzed strains, with the exception of strain MRSA252, in which PSM-α3 carries the previously identified mutation N22Y. This mutation is characteristic of CC30 strains and leads to a significant decrease in both their cytolytic properties in relation to human neutrophils and pro-inflammatory potency. It is noteworthy that CC30 strains exhibit an increased bacterial contamination in the bacteremia model compared to strains in which the mutant *psm-α3* gene in the genome was replaced with an intact one. This mutation is believed to reduce pathogen recognition and allow the bacteria to avoid elimination by innate host defenses during bloodstream infections [90].

##### Superantigens

The identified prophage deletion was accompanied by the loss of three genes encoding IEC1 proteins: SAK, CHIPS, and SCIN, which are present in the genomes of most sequenced *S. aureus* strains. However, the gene encoding enterotoxin A remained intact in the prophage region, which indicates the extreme importance of this pathogenicity determinant. The genes encoding enterotoxin K and enterotoxin-like protein Q were identified in the SaPI1 island fragment, about 3000 bp long. Another member of the enterotoxin family was identified, the RAM47603.1 protein, 234 amino acid residues in size and having only 40.87% identity with enterotoxin A. The protein was identified as a member of the enterotoxin family type 26 in the NCTC 8325 genome and probable enterotoxin A in the Col genome using BLASTp. Enterotoxins are multifunctional proteins that are able to exhibit the properties of superantigens. They are able to cross-link MHC molecules of class II antigen-presenting cells with the T-cell receptor of T-lymphocytes, promoting non-specific proliferation of lymphocytes with subsequent anergy and release of large amounts of cytokines, causing a “cytokine storm” [20,91].

#### 2.3.3. Virulence Factors Involved in Inhibition of Phagocytosis

A unique set of genes encoding superantigen-like proteins SSL1–10, as well as SSL11 and SSL12–14, was identified in the SA943 genome (Supplementary Table S1). A similar set was not found in the other genomes. The role of the products of these genes is ambiguous. By binding to TLR 1/2 and TLR 2/6, they block pathogen recognition (SSL3), disrupt the processes of extravasation, rolling, and chemotaxis of neutrophils, and can cause the formation of vascular thrombi and bleeding (SSL5, SSL6, SSL11) [92,93,94]. The studied genome encodes several proteins that prevent complement activation and phagocytosis of microbial cells, among them the previously mentioned SpA, SSL7, SSL10, complement convertase inhibitor (Ecb), Eap, as well as the second immunoglobulin binding protein—Sbi [30,95,96,97,98] (Supplementary Table S1). The secreted Sbi protein present on the cell membrane contains two IgG binding domains homologous to the B and D domains of protein A and two domains interacting with complement factors C3d and H. This interaction leads to impaired recognition of the B antigen by cells. Acting synergistically with protein A, Sbi manipulates the molecular mechanisms of the innate and adaptive immune response. Together with extracellular fibrinogen-binding protein (Efb), Sbi recruits human plasmin and causes degradation of key complement components C3 and C3b [99,100]. The interaction of Eap with intercellular adhesion molecules 1 (ICAM-1) leads to the inhibition of leukocytes binding to activated endothelial cells, as well as the movement of leukocytes from the bloodstream to the site of infection [101]. Eap inhibits the classical and lectin complement pathways and prevents *S. aureus* opsonophagocytosis and neutrophil killing [102]. It disrupts the interaction of complement components C2 with C4b, prevents the formation of CP/LP C3 proconvertase and C3b formation, blocks the binding of C3b to complement factor B and the formation of active C3 convertase, and also affects the activity of neutrophil serine proteases such as elastase, proteinase 3, and cathepsin G [103,104]. Eap interacts with plasma proteins such as fibronectin, fibrinogen, laminin, and prothrombin and reduces the formation of NETs (neutrophil extracellular traps consisting of DNA-histone scaffolds) [105]. With its C-terminus, it interacts with C3b, and with its N-terminus with fibrinogen, it forms a trimolecular complex that promotes the deposition of fibrinogen on the surface of the microbial cell and causes the formation of a protective pseudocapsule (fibrin network) around the bacteria with the help of prothrombin. Efb induces fibrinogen binding to platelets, disrupts their activation, and, as a result, inhibits the formation of active platelet–monocyte and platelet–granulocyte complexes involved in the innate immune response [106]. The genome also contained *S. aureus* specific genes encoding superoxide dismuteses (SodA/SodM), katalase (KatG), and adenosine synthase (AdsA) proteins that reduce oxidative stress caused by reactive oxygen species and promote the survival of the microbe inside neutrophils [107,108,109]. In the analyzed genome, genes encoding proteins involved in the complex and multi-stage process of *Staphylococcus* agglutination with fibrin, which also provides protection of the microbe from phagocytes, were identified, including the superantigen-like protein SSL10, coagulase (Coa), von Willebrand factor, adenosine–synthase. It has been shown that, in addition to its main function, Coa inhibits proliferation and induces apoptosis of osteoblasts, leading to a decrease in bone formation, an increase in RANCL, and, ultimately, an increase in bone resorption due to stimulation of osteoclasts [110,111].

#### 2.3.4. Characterization and Contribution of Proteases to the Development of the Infectious Process

Most of the *S. aureus* strains have ten major secreted proteolytic enzymes located in four operons. These include a metalloproteinase (aureolysin, Aur), two cysteine proteases: stafopain A (ScpA) and stafopain B (SspB), serine protease V8 (SspA), and six serine-like proteases that are homologues of SspA (SplABCDEF) [112,113,114,115]. Despite some inconsistency of research data on determining the contribution of extracellular proteases to the infectious process, convincing evidence has been obtained for the participation of these enzymes in immune evasion processes as a result of the various mechanisms of phagocytosis inhibition and survival inside macrophages [116]. With high enzymatic activity, secreted proteases can cleave human α1-proteinase, α1-antichymotrypsin inhibitor, heavy chains of all classes of human immunoglobulins, elastin, fibrinogen, fibronectin, collagen, high molecular weight kininogen, and plasminogen [117,118,119,120]. The participation of cysteine proteases, in particular ScpA, in the processes of IgG proteolysis, impaired migration of lymphocytes to the site of infection, and induction of endothelial cell killing has been shown [118,121,122,123,124]. Aur can protect staphylococci within phagocytes, probably due to protection against killing by antimicrobial peptides [125]. It is curious that the shutdown of these enzymes action can lead to an aggravation of the infectious process. Thus, under experimental conditions, there is an increase in the expression of cytolytic toxins, including alpha-toxin, HlgAB, HlgCB, PSMs, LukE, LukA/B, PVL, a number of cell-associated proteins (fibronectin-binding proteins, clumping factor A, Sbi), as well as some other pathogenicity factors in a protease-free strain compared to the wild-type strain [126,127]. It has been demonstrated that protease null mutant cells have an increased cell wall thickness due to a higher concentration of surface proteins, while the protease null strain exhibits greater adhesive ability to surfaces covered with plasma proteins, as well as elastin. In addition to interacting with the host organism, secreted proteases can modulate the stability of their own pathogenicity determinants. In particular, SspA has been shown to cleave surface proteins, including fibrinogen-binding protein and protein A. In addition, Aur cleaves surface-bound cohesion factor B proteins [126]. Cleavage of these proteins by extracellular proteases is believed to influence the transition from an adhesive to an invasive phenotype. It has also been suggested that extracellular proteases can cleave secreted toxins and, thus, regulate the abundance of virulence factors depending on the presence of the pathogen in a particular host niche [127,128]. Serine-like proteases Spl A–F are encoded by a single operon as part of the vSaβ pathogenicity island [114,129]. Despite the different substrate specificity, they show a high degree of amino acid composition similarity, both among themselves and with the ScpA protease and exfoliative toxins [130,131,132]. Their role, apparently, is not limited only to providing the nutritional needs of the microorganism, but is determined by the peculiarities of their pathogen–host interaction. They are reported to be involved in allergic processes, promote pathogen dissemination, and aggravate the course of pneumonia. SplD can induce IgG 4/IgE antibodies in humans, induce the expression of a type 2 cytokine immune pattern, and contribute to the development of asthma [133,134]. Twelve proteases have been identified in the SA943 genome (Supplementary Table S1), among them the widespread three cysteine proteases (staphopain A, B; staphostatin B) and the zinc-dependent metalloprotease Aur. Unlike other members of ST239 (Tw20, JKD 6008, T0131, Z172, V521), SA943 has 498 amino acid residues in Aur due to the deletion of the first 12 amino acids. The serine-like protease operon included SplA, B, C, and SplF, following a 1744 amino acid deletion. SplE and SplD were lost. In addition, rarer and still insufficiently characterized serine proteases have been identified, including the intramembrane serine protease RAM47553.1 (487 aa), which belongs to the family of intramembrane rhomboid proteases. This protease is highly conserved and has been found in the genomes of strains of various sequence types (Col, Newman, N315, 55/2053, TW20, T0131, Z172, Bmb 9393). An unusual, little-studied serine protease CtpA (C-terminal processing peptidase) of 496 aa, belonging to the S41 family of carboxy-terminal peptidases, which are involved in the C-terminal cleavage of proteins, has been identified. The role of this protease in maintaining cell wall stability, mechanisms of bacterial stress resistance, and protection against components of the host immune system was demonstrated [135]. A conserved amino acid sequence specific for CtpA was present in the ST8 and ST239 genomes. Finally, the unique membrane-bound serine protease RAM47657.1, which, according to BLASTp results, was present only in the genomes of ST239 representatives isolated in Turkey. The set of peptidases also included peptidase S8 (RAM 45670.1), which is believed to be involved in the degradation of collagen, casein, and the C-terminal cleavage of IL-8 [115].

### 2.4. Regulation of Virulence

The expression *S. aureus* toxins is tightly controlled by a regulatory network that includes several regulators, including Agr, SarA, and SaeRS [32]. All of them are required for alpha toxin expression, but only AgrA and SarA affect PSM expression. The experimental results show that the virulence determinants responsible for the osteoblasts’ death after invasion are under the control of the regulatory loci AgrA and SarA, but not SaeRS, which is consistent with the main role of PSM in intracellular virulence [24,78]. Since both Agr and SarA can suppress the transcription of protein A, a key pathogenicity factor in the development of osteomyelitis, it seemed appropriate to analyze the amino acid sequence of proteins of both loci and try to identify the possible presence of mutant variants.

#### 2.4.1. Agr Regulon

The Agr locus encodes a two-component recognition system for the size of a microbial population or quorum, which is based on the formation of two divergent transcribing products, RNAII and RNAIII. The RNAII transcript is encoded by a four-gene operon, *agrBDCA*. AgrC and AgrA correspond to the sensor and activator of a two-component regulatory system. AgrB and AgrD are involved in the synthesis of cyclic octapeptide (AIP), which acts as a quorum-sensitive molecule [136,137]. With extracellular accumulation of a critical concentration of the cyclic octapeptide, the AgrC sensor protein is phosphorylated, which leads to the second stage of phosphorylation, in which AgrA is phosphorylated. Phosphorylated AgrA activates the transcription of RNAIII, an effector molecule, the formation of which leads to the activation of the synthesis of extracellular proteins (for example, Hla) and the suppression of the synthesis of proteins associated with the cell wall (for example, SpA and fibronectin-binding proteins). In addition, Agr makes a significant contribution to the biofilm functions [138]. It should be noted that AgrA activation can also occur in a limited space. It has been shown that, after phagocytosis, RNAIII expression can be activated inside a eukaryotic cell, even if only one bacterium is present in the phagosome [139]. Differences in the Agr locus are based on the sequence variation in AIP, its AgrB processor, and the AgrC receptor, which forms specific functional units and works in concert. Based on these variations, four Agr specificity groups are distinguished. Recent discoveries show that RNAIII regulates many target genes through the control of a repressor protein gene called Rot, a member of the SarA family of transcription regulators [140,141]. AgrA can directly control the expression of α- and β-PSM, independent of RNAIII, through a yet unknown mechanism. Importantly, the Agr defect correlates with increased duration and mortality due to bacteremia during antibiotic treatment and with a greater incidence of glycopeptide antibiotic resistance than in strains with an intact locus [142,143].

Among the proteins of the Agr locus, the greatest variability in the amino acid sequence was found in AgrC (Supplementary Table S8). The protein size ranged from 233 to 430 amino acid residues. The sequence in strain Col with a length of 430 aa was considered as a reference. In the secondary structure of the protein, a transmembrane domain (1–200) is isolated, and a cytoplasmic domain, in turn, consisting of two subdomains: dimerization and histidine phosphorylation (DHp: 201–300), as well as a catalytic ATP-binding (CA: 301–430) [137,144]. SA943, unlike most strains of ST239, had a protein of 414 amino acid residues as result of a deletion of 16 amino acid residues at the C-terminus, similar to strain NCTC 8325. Three amino acid substitutions were identified, two of which were in the CA subdomain sequence: I311T, A343T. Unlike the Col strain, SA943, as in all other analyzed strains, also had the 247P/T substitution in DHp. It should be noted that in the T0131 genome closest to SA943, the length of AgrC was 233 amino acid residues as a result of the loss of the entire transmembrane domain and a partial deletion of the DHp subdomain. The greatest differences in the AgrC sequence, characterized by numerous substitutions, were found in strains N315 (agr2), MW2, MRSA252, and 55/2053 (agr3). At the same time, the N315 strain had both an incision (1–7) at the beginning of the transmembrane domain, a subsequent deletion up to 66 amino acid residues, and a deletion of 13 amino acids in the CA subdomain, which led to a shortening of the protein sequence to 371 amino acids. The amino acid sequences of the AgrA, AgrB, and AgrD proteins in the SA943 strain were highly conserved and showed 100% homology with both the Col strain and the NCTC8325 strain, as well as with most of the ST239 strains. The variability of the amino acid sequence of AgrB and AgrD proteins in other analyzed strains correlated in accordance with their belonging to different Agr groups.

#### 2.4.2. SarA Family Proteins

Unlike Agr, the SarA locus activates the synthesis of both extracellular (eg, HlgAB, and HlgCB) and cell wall-associated proteins (eg, FnbA) [145]. The most studied representative of the locus is the SarA DNA-binding protein, which consists of 124 amino acid residues. SarA can regulate target genes by binding directly to their promoters or indirectly through downstream effects on regulons (e.g., binding to the Agr promoter) or by stabilizing mRNA during the logarithmic phase SarA, which binds to a 29 bp recognition sequence within the interpromoter P2–P3 region of the Agr region, playing a significant role in the activation of Agr transcription. Eight SarA homologues, collectively referred to as the SarA protein family, have been identified [146,147]. Depending on the size, the SarA protein family can be divided into three subfamilies: (1) single domain proteins (SarA, -R, -T, -V and -X, and Rot); (2) two-domain proteins (SarS, -U, and -Y); and (3) MarR homologues (MgrA and SarZ). The proteins of this group form a complex mutually subordinate network of regulation, which can also interact with the products of the Agr locus [147,148,149]. The amino acid sequences of proteins encoded in the Sar locus turned out to be highly conserved (Supplementary Table S9). Almost all proteins encoded in this region (SarA, SarR, SarS, SarT, SarX, SarV, SarU) were detected in SA943, with the exception of SarZ, most of which showed 100% homology with the reference sequences. The greatest variability was shown by Rot proteins. The size ranged from 133 amino acids in *S. aureus* NCTC 8325 and, in most of other strains, to 166 aa in *S. aureus* Col, MRSA252, and N315. However, these 133 amino acids were identical in all strains, including SA943. 

### 2.5. Characteristics of the Proteins Involved in Capsule and Biofilm Formation

The genes whose products are involved in the biosynthesis of the type 8 capsule and in the formation of the polysaccharide matrix of biofilms (icaADBC, icaR), which protect the microorganism from phagocytosis, were identified in SA943 genome (Supplementary Table S1). However, it is known that the acquisition of resistance to methicillin represses the formation of the biofilm polysaccharide matrix and promotes the formation of a protein biofilm type [149]. SA943 contains a whole set of genes that form the protein base of the biofilm matrix and are involved in the processes of microbial cell adhesion, biofilm maturation, its dispersion, and dissemination of planktonic bacteria. Among them, the key role belongs to FnBPA, SdrC, SpA, SasG, and Eap [150,151]. It has been shown that FnbA and SdrC are involved in attachment to both biotic and abiotic surfaces and carry out intermicrobial interactions at the stage of biofilm maturation. It is believed that FnBPs induce biofilm formation by a mechanism based on multiple, Zn^2+^-dependent, hemophilic, low-affinity bonds between FnBPA or FnBPB A-domains located on neighboring cells [42,152]. The involvement of protein A in biofilm formation is characterized by several hypotheses. According to one of them, protein A-mediated aggregation and biofilm formation may be the result of homophilic interactions between two molecules of protein A of neighboring cells. Alternatively, protein A can provide heterophilic interactions with other surface proteins or even with non-protein components of the cell wall. It has been shown that the covalent attachment of protein A to the bacterial surface is not required for its ability to carry out intercellular interactions. A secreted protein A or protein A variant lacking the carboxy-terminal LPxTG domain is sufficient to induce biofilm development. In contrast to the mechanisms of Aap- or SasG-mediated biofilm development, which require activation of these proteins through proteolytic processing to participate in intercellular interactions, protein A induces biofilm development in the Agr mutant, which produces low levels of proteases [153]. The major autolytic protein (AtlA) is the key enzyme that releases extracellular DNA, another major component of the *S. aureus* biofilm matrix [151]. Eap plays a key role in the rigidity of the biofilm structure, while SasG binds to extracellular DNA and stabilizes it [154]. Cytolytic toxins such as Hla and Hlb also contribute to the formation of the biofilm matrix. It is assumed that Hla takes part in the formation of intercellular interactions at the initial stages of biofilm formation, while Hlb, due to the presence of ligase activity, participates in the formation of the skeletal nucleoprotein matrix of the biofilm, forming covalent cross-links between its molecules in the presence of extracellular DNA [89,155,156]. Extracellular proteases, as well as Hld and other PSM peptides, which have the properties of surfactants, also play a significant role in the process of biofilm dispersion. PSM are involved in the processes of biofilm structuring, channel formation, detachment, and dissemination of planktonic bacteria inside the macroorganism [157,158]. Activation of Agr also promotes the detachment of microbial cells from the extracellular matrix of a mature biofilm and further distribution in the internal environments of the host [138].

## 3. Discussion

*Staphylococcus aureus* is a widespread pathogen, unique in its properties, capable of not only colonizing from 20 to 30% of people in a population but also causing diseases in almost all human organs and systems. The increasing frequency of isolation of multiple antibiotic resistant MRSA strains significantly limits chances of effective antibacterial therapy for staphylococcal diseases and facilitates the development of chronic infection. At the same time, relapses of the disease are observed approximately four times as often as infection with a new strain [159]. The aim of this study was to identify features of the SA943 genome that contribute to the formation of various types of infectious process. For comparison, we used annotations of genomes of *S. aureus*, namely, seven strains of the most epidemically successful sequence types, both methicillin-susceptible and hospital and community-acquired MRSA, including strains belonging to different subclones of a HA-MRSA ST239. ST239 is an epidemic clone of MRSA, which has the largest number of antimicrobial resistance genes and mechanisms, which provide it with evident advantages for distribution in the hospital environment. SA943 was found to be resistant to eight classes of antimicrobials. In addition to the amino acid substitution in the PBP-2a protein, which requires MIC of the fifth-generation cephalosporin (ceftaroline fosamil) at 2 µg/mL, during persistence, it underwent an amino acid substitution in the cell wall PBP2, which increased the MIC of the antibiotic to 4 µg/mL. Like other strains of ST239, SA943 has a high level of methicillin resistance (MIC ≥ 256 µg/mL). It is known that an increase in the expression of resistance to methicillin leads to suppression of the regulation of the Agr system [149]. It can be assumed that a further increase in resistance to beta-lactam antibiotics leads to even greater suppression of this regulatory system.

As with other analyzed ST239 genomes, the SA943 genome has a number of key features that reduce its virulence. All ST239 genomes, unlike MRSA strains of other sequence types, contain SCC*mec* III, which, on the one hand, carries the *mecA* that encodes for PBP-2a, the key molecule responsible for resistance to beta-lactam antibiotics, and on the other hand, it carries the gene encoding the phenol-soluble modulin (PSM*mec*). A high level of *mecA* expression in a number of hospital-acquired MRSA strains induces changes in the cell wall that affect the Agr quorum sensing system, which ultimately leads to a decrease in synthesis of cytolytic toxins [160]. Another mechanism for attenuating the virulence of MRSA ST239 is the suppression of Agr translation through the action of *psm-mec* srRNA. It has been shown experimentally that introduction of the *psm-mec* into FRP3757, strain CA-MRSA USA300 carrying SCC*mec* IV (does not contain *psm-mec*), or Newman, a methicillin-sensitive *S. aureus* strain carrying neither SCCmec nor *psm-mec*, reduces the amount of secreted PSMα, inhibits the colony growth, and promotes the biofilm formation. Strains transformed with *psm-mec* have reduced virulence in a mouse model of systemic infection [85,161,162]. Alternatively, *psm-mec* srRNA transcription activity can increase the expression of SpA, one of the important *S. aureus* proteins responsible for bone tissue destruction and the development of osteomyelitis [90]. A peculiar structural organization of the gene encoding SpA is a unique common feature of ST239 genomes. As it appears, the *spa* in all analyzed ST239 genomes contains four functional domains, instead of five, as in the reference genome of the *S. aureus* NCTC 8325 strain. Experimental deletion of gamma globulin binding domains of NCTC 8325 revealed that a smaller number of domains may diminish strain virulence [48]. These findings were confirmed by a study of clinical isolates of ST239 [163]. However, a smaller number of domains and formation of a recombinant domain, leading to conformational changes in the protein, apparently do not cause a decrease in the level of SpA expression in ST239 strains. Furthermore, SpA expression is increased in clinical isolates of HA-MRSA ST239, both at the RNA and protein levels, compared to highly virulent CA-SA ST398 isolates [164]. Nevertheless, it can be assumed that a smaller number of functional domains reduces the pathogenic potential of the pathogen, reducing the number of host molecules with which it is able to interact. SpA has been shown to recognize TNF-α receptors on the surface of both epithelial cells and osteoblasts and induce inflammation through the TNF-α-TNFR1 signaling pathway, resulting in neutrophil recruitment and activation, sometimes at the cost of neutrophil damage to surrounding tissues [101]. Among inducible pro-inflammatory cytokines, TNF-α is critical for bacterial eradication [165]. However, the early release of TNFR1 can neutralize circulating TNF-α, attenuate the host’s inflammatory response, interfere with bacterial clearance, and promote long-term microbial colonization. A change in the molecular structure of SpA appears to affect the ratio of its soluble to its cell-bound forms, which is a decisive factor in evading the immune defense of the host during *S. aureus* infection [165]. In addition, MRSA ST239 strain has a higher ability for internalization and persistence in the cell culture of osteoblasts compared to the common, in Italy, osteomyelitis-associated MRSA-SCC*mec* I ST228 strain [166].

Notwithstanding, a higher infective dose of *S. aureus* ST239 is required to reproduce an acute infection in mice, compared to *S. aureus* ST398 and ST30 [34,164]. Undoubtedly, it cannot be ruled out that other pathogenicity factors present in the *S. aureus* strains selected for comparison could contribute to the development of the infectious process. However, the above data suggest that SpaA is not a key virulence factor in acute *S. aureus* infections, but may contribute to long-term damage in a host infected with HA-MRSA ST239 [164]. Recently, a previously unknown mechanism of damage by SpA has been discovered, which does not require interaction with receptors on the surface of a eukaryotic cell. It has been shown experimentally that in the presence of human serum, toxic SpA–IgG complexes are formed, which are capable of causing necrosis not only of B cells but also of monocytes. Vaccination of mice with sera induced by the non-toxigenic mutated SpA is able to suppress this mechanism [167,168]. For the manifestation of the superantigenic activity of SpA against B cells, it is necessary that the SpA molecules contain intact domains of the LysM and LPxTG motifs with associated peptidoglycan fragments. The LysM domain binds glycan chains of peptidoglycan fragments, while the LPxTG motif is covalently bound to wall peptides that lack glycan. These results highlight the complexity of SpA interactions with B cell receptors. It is the LysM domain associated with peptidoglycan glycan strands that is thought to influence certain B cell signals that deflect pathogen-specific adaptive immune responses [169]. Sa943, like T0131, contains the amino acid substitution of aspartic acid for alanine in the LysM region (D461A), the functional significance of which remains unexplored.

A whole set of genes has been identified in the SA943 genome, whose products provide the ability to resist the host immune system. The pathogen is able to inhibit neutrophil extravasation, activation, and chemotaxis with the help of members of the SSL family, including SSL3, SSL4, SSL5, and SSL10 [32,92,94,98]. Such proteins as protein A, Sbi, and SSL10 are able to interact nonspecifically with the Fc region of IgG, disrupt the deposition of IgG on the surface of bacteria, and prevent their effective opsonization [99,100]. Genome Sa943 encodes a number of proteins that can successfully compete with the components of the complement system, including Cna, which blocks the classical pathway, SdrE—alternative pathway, and Eap—lectin and classical pathways [67,73,102]. In addition to them, the fibrinogen-binding protein Efb promotes the deposition of fibrinogen on the surface of the microbial cell and, thus, prevents the recognition of C3b, the key component of the complement system, on the surface of the microbial cell. SSL7 interacts with the Fc fragment of IgA and the C5 component of complement and prevents the bacteria death under the action of serum components [97]. The process of inhibition of neutrophil killing can be carried out by the action of such products as staphyloxanthin, superoxide dismutase, catalase, lactate dehydrogenase, and staphylococcal peroxide inhibitor [32]. Killing resistance and survival within neutrophils and, especially, macrophages are ways in which a pathogen takes advantage of the native immune response and can promote bacterial spread by making phagocytic cells become containers for the spread of the microbe in the host [125,170,171]. The deletion of most of the SD repeats of the SdrE protein, which blocks the alternative complement pathway, was the original mechanism of immune evasion implemented in the SA943 genome. During infection, SD residues undergo glycosylation, the intensity of which depends on the number of available SD repeats. Insertion of additional residues stimulates antibodies to recognize these proteins, while deletion of SD repeats may be a mechanism used to evade host immune surveillance. Genetic variation in this region may indicate adaptation of *S. aureus* to the environment without loss of functionality of other regions within the protein [172]. At the same time, it should be noted that another member of the Sdr family, namely SdrD, in SA943 turned out to be highly conserved and contains the fixed number of SD repeats, which once again indicates the different contribution of these proteins to the virulence of *S. aureus.* Invasion of *S. aureus* into organs and tissues from the bloodstream requires not only immune evasion, but also adhesion and further structural changes within the eukaryotic cell. SA943 has such significant members of the MSCRAMMS family that perform these tasks, such as Cna and FnBpA. Invasion into nonprofessional phagocytes, which include osteoblasts, is ensured by at least three proteins present in SA943: SpA, FnBpA, and Eap. In order for the microorganism to be able to exit the phagosome, the production of PSMs is necessary, but apparently not sufficient. It has been shown that in addition to PSMs, beta-toxin, whose function is restored in SA943 due to loss of part of the prophage, alpha-toxin, and leukotoxin G/H, is actively involved in these processes [21,22,88,173]. The development of a chronic or recurrent infection caused by *S. aureus* indicates a defective humoral and T cell memory response. The SA943 genome encodes the leukotoxin LukE/D, which can cause the death of not only neutrophils but also immune memory cells, four enterotoxins that have superantigenic activity against T cells and take advantage of the immune response to conventional antigens, the extracellular adhesion protein Eap, which also weakens cellular immunity by reducing the proliferation of T cells, and delta-hemolysin, which has cytolytic activity in relation to T cells and also triggers degranulation of mast cells. The presence of functionally active SpA, with superantigenic activity against B cells, leads to immunodominance of this protein, subverting host responses to other *S. aureus* virulence factors, necessary for protection and formation of immunological memory [174]. The events that lead to intracellular persistence as opposed to cell lysis are not yet fully understood. It is possible that this process involves SCV cells, which have a reduced metabolism and do not express cytolytic toxins. SCV formation is under the control of SigB and is critical for *S. aureus’* adaptation during chronic infection [175]. The dualism of SA943 was clearly manifested in the fact that it retained a whole arsenal of genes for adhesins, cytolytic toxins, various proteases, and other biologically active substances necessary for the development of an acute infection, which ultimately allowed it to switch from the invasive phenotype to the aggressive phenotype. Most of the aforementioned key proteins are highly conserved; the amino acid substitutions in the protein sequence of ST239 do not affect the ligand-binding domains, but are localized in the variable regions. Like other virulent strains, SA943 contains a set of genes encoding cytolytic toxins. The products of these genes in most of the analyzed strains are highly conserved, with the exception of gamma-hemolysin subunit C, which, in strains N315, MW2, MRSA252, and 55/2053, has numerous amino acid substitutions. A feature of SA943, as well as other ST239 genomes, is the presence of an amino acid substitution in the Hla signal sequence. The present study did not assess the level of expression of this toxin in SA943; however, apparently, it can be quite high, at least in some representatives of this clade [166]. The Sa943 genome encodes a wide range of PSMs, including PSMmec, which, in addition to its main function, regulates the expression of pathogenicity factors by repressing the Agr system. The role of PSMs in the pathogenesis of both acute and chronic infections is multifaceted [85,90]. PSMs are able to induce cytotoxicity in host cells through receptor-independent pore formation. These peptides promote the release of captured microbial cells from osteoblasts and have a damaging effect on the latter, which hampers bone remodeling processes [86,87]. By activating neutrophils through the formyl peptide receptor (FRP-2), they induce the synthesis of pro-inflammatory cytokines. In bacteremia, PSMs together with Hla are able to cause a systemic increase in the concentration of IL-6, which is an activator of osteoclasts and facilitates the destructive bone resorption by increasing the number and activity of osteoclasts [176]. In addition to participating in the processes of avoiding intracellular digestion, PSMs play an important role in the processes of structuring and dispersion of biofilms [157]. The revealed high conservatism of PSMs in most of the studied strains indicates their important role in the pathogenesis of staphylococcal infection. Developing in the macroorganism, the Sa943 genome has undergone a number of changes, most of which are aimed at further reducing virulence and enhancing the ability to form biofilms. Sa943 has lost most of its prophage DNA and, unlike such highly virulent ST239 representatives as TW20 and T0131, does not carry any intact prophage known to be able to enhance the virulence of the microorganism [163,177,178]. However, at the cost of the partial loss of the nucleotide sequence of the beta-converting prophage, along with the loss of such significant pathogenicity factors as staphylokinase, CHIPS and SCIN proteins, function of Hlb, an active participant in the release of phagocytosed microbial cells into the cytosol and the death of eukaryotic cells, was restored. Recent data indicate that Hlb is an important virulence factor. In addition to the previously mentioned functions, Hlb has anti-angiogenic properties that can not only impair inflammatory signaling in endothelial cells, but also prevent proper vascular repair, keeping the endothelium in pro-inflammatory, hypercoagulable state, as well as preventing the processes of healing and formation of new vessels, which can play a significant role in the pathogenesis of osteomyelitis [179,180]. The loss of prophage (or a part of prophage) is considered a form of active lysogeny, in which the excision of phage functions as a regulatory mechanism for the expression of bacterial chromosomal genes, while the released phages do not enter the lytic cycle and do not form plaques on the host strain. The ϕSa3int prophages act as new phage regulatory switches (phage-RS), providing conditions for Hlb expression and promoting molecular mechanisms of adaptation to environmental conditions in the host organism. [181]. Of note, another very important factor of immune evasion, the staphylococcal complement inhibitor (SCIN), localized on the bacteriophage, was lost. SCIN inhibits all three complement pathways: alternative, classical, and lectin. Its mechanism of action is based on the stabilization and inhibition of surface-bound C3 convertase, which leads to a decrease in C3b deposition and the release of the C5a chemoattractant and blocking phagocytosis. SCIN-B and SCIN-C also inhibit complement [30]. SCIN-B has been identified in the SA943 genome (Supplementary Table S1), that can perform the functions mentioned above. SA943 has a biofilm and capsule gene cluster that also allows it to resist antibiotics and phagocytosis (Supplementary Table S1). The pathogen secretes its own coagulase, causing the deposition of fibrin on the surface of host cells. Using immunostaining, it was found that in addition to bacterial cells and their products, the composition of the biofilm matrix also includes human serum proteins, including fibrinogen, which is involved in the formation of the structural framework of the biofilm. It has been proven that under the action of staphylokinase-activated plasminogen, the fibrin skeleton of the biofilm matrix is dissolved, which significantly increases the susceptibility of biofilms to antibiotics and phagocytosis by neutrophils [182]. Both laboratory and clinical Sak-free strains of *S. aureus* have been shown to form thicker biofilms than high Sak-producing strains [183]. SA943 has lost SAK, which gives it additional advantages in biofilm formation. Significant changes affected the regulatory locus Agr. Unlike most strains of ST239, SA943 carries a truncated AgrC as a result of a 16-amino acid deletion at the N-terminus, similar to strain NCTC 8325, and contains 3 amino acid substitutions, 2 of which were located in the CA subdomain—I311T, 343 A343T. Experimental evidence has now been obtained that naturally occurring mutations in the cytoplasmic domain of AgrC, including T247I, I311T, and A343T, are associated with reduced cytotoxicity, delayed or decreased production of AIP, and impaired sensitivity to exogenous AIP, due to repositioning of key functional domains, impaired dimerization processes, and restricted access to the ATP-binding pocket. The result of these events is an increased threshold for Agr activation through AIP-dependent autoinduction, and thus a reduced virulence that keeps *S. aureus* in “colonization” mode [184]. The consequence of the inactivation of the Agr system is overexpression of protein A and an increased ability to form biofilms. Unlike Aap or SasG, whose participation in intercellular interactions requires the activation of these proteins through proteolytic processing, protein A is able to induce biofilm development in the Agr mutant, which produces low levels of proteases [154]. Covalent attachment of protein A to the bacterial surface is not required to induce biofilm development and intercellular interactions; the presence of a secreted SpA or even a protein variant lacking the LPxTG motif is sufficient [153]. The tendency towards chronicity of bone infections and joints, as a result of dysfunction of the Agr system, may be the result of a decrease in the production of delta-toxin, enhanced microbial cell internalization, reduced cytotoxicity of the pathogen in relation to osteoblasts, and increased ability to produce biofilms, but not to form SCV [152]. It has been shown that Agr-negative strains have an adaptation advantage compared to Agr-positive strains in the presence of sublethal concentrations of some antibiotics, and that lower fitness of Agr-positive strains is caused by antibiotic-mediated expression of the Agr effector molecule, RNAIII [143]. In vitro experiments have shown that, as a result of serial passages, some Agr-negative strains can revert to the wild type, i.e. the original Agr activity. Furthermore, Agr-negative planktonic cells can revert to Agr-positive within the phagosome. Possibly, phagocytosis induces a certain signal, supported by the environment of *S. aureus*, for AIP to accumulate inside the phagosome [139]. It is believed that bacteria can resort to Agr phase variations as a covert strategy for maintaining the infectious process, while maintaining the ability to survive phagocytosis [185]. SA943 contains conserved, and therefore functionally active, proteins encoded by the Sar locus, whose notable role is also participation in biofilm formation. In addition, suppression by SarA of extracellular production of proteases and nucleases that are capable of destroying PSM results in an increase in the concentration of PSM, which, in turn, can enhance the death of both osteoblasts and osteoclasts.

In the structure of the vSaβ pathogenicity island, a 1744 bp deletion was detected, which led to the loss of SplE and SplD. It has been shown that the activity of SplD and SplF can provide the pathogen with certain advantages in the pathogenesis of osteomyelitis [186]. The loss of some of the proteins of the spl operon, which have a high degree of similarity in amino acid composition, apparently does not significantly affect the pathogenic potential of SA943. Moreover, the SA943 genome contains additional serine proteases, including the very rare protease RAM47657.1, which is present only in the genomes of some members of the Eurasian subclone ST239 (*S. aureus* Hu14, 15, 16, Deu 3, 5, 6, 8, 12, 16, 17) isolated in Turkey [187]. These data serve as an additional confirmation of our earlier evidence of a close genetic relationship between SA943 and strains isolated in this region [36]. The main function of *S. aureus* extracellular proteases is to control the progression of infection by selectively modulating the stability of virulence factors. Seven main elements (SarS, SarR, Rot, MgrA, CodY, SaeR, and SarA) form the main control network for the expression of protease operons, with the last three being the most efficient. Aur expression is largely repressed by these factors, while the spl operon is highly upregulated by any of the regulatory loci listed, but especially by SarR and SaeR, which remained intact in SA943. On the contrary, when studying ScpA expression, it was found that the named loci affect it in the opposite direction: SarA (repressor) and SarR (activator). Seven additional factors (ArgR2, AtlR, MntR, Rex, XdrA, Rbf, and SarU) have been identified that form a secondary chain of protease control [188,189]. A complex two-stage system of genetic regulation of the expression of these pathogenicity factors, including many SarA regulon loci, as well as some other genes, indicates their important role in the development of the infectious process. Not surprisingly, this group of genes remained intact in SA943. The dual role of proteases in the development of the infectious process is emphasized by the following observations. Protease-deficient microbes are hypervirulent, and mice infected with such a mutant show a dramatic reduction in survival in a septic infection model [74]. However, the double mutant in the Agr-regulated metalloprotease Aur and serine proteases, SplABCDEF, shows minimal extracellular protease activity, improved biofilm formation, and a highly attenuated detachment phenotype [138]. 

It is clear that *S. aureus* can modulate its growth and virulence in response to different environments. The limitation of this study lies in the fact that the analysis focused on studying the features of the set and molecular structure of those *S. aureus* pathogenicity factors that had been previously associated in one way or the other with the development of osteomyelitis. However, applying the TnSeq technology to a model of osteomyelitis in mice Wilde, AD et al. (2015), more than 200 major genes were identified and the staphylococcal genetic and metabolic programs required to maintain an invasive infection were characterized [186]. This analysis suggest that the development of osteomyelitis is accompanied by significant changes in the physiology of the pathogen, affecting not only the pathogenicity factors, but also the processes of metabolism and energy production in low oxygen conditions in the bone tissue.

In summary, a number of common specific genetic features that could affect the virulence MRSA ST239 clade have been identified, namely, the change in the structure of protein A, presence of PSM*mec*, and amino acid substitution in the Hla amino acid signal sequence. Some of the identified changes were found only in representatives of the Eurasian subclone. Some of the changes were specific for SA943 genome only. For the first time, we revealed the four-domain structure of the A protein, which turned out to be a specific characteristic of the representatives of MRSA ST239. We found that this event was not simply the loss of one of the protein domains, but was the result of the formation of a recombinant domain carrying numerous amino acid substitutions, which can undoubtedly lead to conformational changes in the protein molecule. We revealed the presence of an amino acid substitution in the LysM domain of SpA, which turned out to be specific only for representatives of the Eurasian subclone. The molecular mechanisms of adaptation that contributed to the development of a persistence infection with SA943 were the appearance of an additional mutation that provides resistance to ceftaroline, the loss of a large part of prophage DNA, and the restoration amino acid sequence of Hlb—an active participant in the processes of exit of phagocytosed bacteria from the phagosome and formation of biofilms; dysfunction of the AgrA system both due to the presence of *psm-mec* in SCC*mec* III, which is involved in the inhibition of translation of the AgrA locus, and the presence of several amino acid substitutions in the AgrC sequence. In addition, we have identified deletion of a part of the nucleotide sequence of the vSAβ genomic island, which led to the loss of two proteases of the Spl operon: SplE and SplD. As a result of the study, a unique set of serine proteases in SA943 genome was identified, which, based on the literature data, can also be an active participant in cross-talk between pathogen and host. It was revealed the original mechanism of immune evasion in one of the Sdr locus proteins, namely, deletion of SD repeats in amino acid sequence of SdrE, a protein that can block the alternative pathway of the complement system. The dualism of SA943 was clearly manifested in the fact that it retained a whole arsenal of genes for adhesins, cytolytic toxins, various proteases, and other biologically active substances necessary for the development of an acute infection, which ultimately allowed it to switch from an invasive phenotype to an aggressive phenotype. Unlike most studies performed in silico, the data obtained in this study were discussed with the involvement of a large number of experimental results obtained by other researchers and confirming the significance and direction of the changes identified at the genomic level.

Nevertheless, in future studies, the role of structural changes in SpA, which has superantigenic activity against B cells and leads to the immunodominance of this protein, should be studied. *S. aureus* Sa943, like T0131, contains, in the LysM region, the amino acid substitution of aspartic acid for alanine (D461A), the functional significance of which remains unexplored. Of great interest are studies on lysogenization of SA943 by beta-converting prophages carrying a different set of immune escape cluster genes. Such experiments will confirm the significance of Hlb in the processes of the bacterial exit from the phagosome and biofilm formation.

## 4. Materials and Methods

*Staphylococcus aureus* strains: Methicillin-resistant *S. aureus* 0943-1505-2016 (SA943) was isolated in 2016 from of a fistula secretion that appeared in a patient 3 years after surgery for osteomyelitis of the vertebral bodies and intermuscular abscess of the surrounding tissues. It is known that during the bacteriological analysis of the pathological material isolated at different stages of surgery from the patient, MRSA strains were isolated repeatedly, which were sensitive only to a few numbers of antimicrobial drugs. Despite the antimicrobial therapy, the eradication of the pathogen did not occur, which led to a relapse of the disease. Since we did not have *S. aureus* isolated from this patient earlier, fully annotated genomes *of S. aureus* strains of several sequence types, both hospital and community-acquired, isolated for more than 60 years, were selected from the NCBI gene bank for comparison.

The representatives of the widespread epidemic clone CC239 belonging to various subclones according to the classification of Monecke S. et al., 2018 [35] were also included (Table 1). It is known that MRSA strains of the USA300 linage (*S. aureus* FPR3757) are highly virulent, capable of spreading both in the out-of-hospital environment and in hospitals, *S. aureus* MW2 is a pathogenic community-acquired MRSA, and Newman is a highly pathogenic methicillin-sensitive *S. aureus* strain [190,191,192]. All three have high virulence in various experimental models. The strains *S. aureus* N315 and NCTC8325 were isolated from patients who died of staphylococcal infection [193,194]. The *S. aureus* MRSA252 is a less virulent strain that caused multiple nosocomial infection outbreaks in UK hospitals in the early 2000s [39]. The representatives of MRSA ST239 belonging to various subclones were isolated throughout the years 1993–2011 in various geographically distant countries of the world. Among them, *S. aureus* TW20, a virulent and highly transmissible strain isolated from a patient in the ICU in the UK, is one of the first *S aureus* ST239 strains to have its genome completely sequenced [163,195]. *S. aureus* T0131 was isolated in China from an 87-year-old patient with bacteremia [35,78]. 

The determination of sensitivity to antibiotics was carried out using a semi-automatic bacterial analyzer BD Phoenix (USA) and by the method of manual microdilutions in broth when determining sensitivity to ceftaroline. Minimum inhibitory concentration values were interpreted according to the EUCAST criteria [196]. Genotyping was performed by multilocus sequencing (MLST) according to (http://saureus.mlst.net (accessed on 17 May 2017)) and single-locus typing based on the determination of the structure and number of variable fragments of the spa gene according to the protocol (http://www.spaserver.ridom.de (accessed on 25 January 2018)), according to Sanger using an ABI 3730 capillary sequencer (USA). Whole genome sequencing was performed on an Illumina HiSeq 2500 platform. The resulting reads were assembled into scaffolds using CLC Genomics Workbench v.7.0 software and SPAdes v.3.11.1. Scaffolds were annotated using RAST (http://rast.nmpdr.org (accessed on 16 March 2020)) and NCBI Prokaryotic Genome Annotation Pipeline (PGAP; https://www.ncbi.nlm.nih.gov/genomes/statistic/Pipeline.htlm (accessed on 24 June 2018) resources. Plasmid search was carried out using the PlasmidFinder (https://cge.cbs.dtu.dk/services/PlasmidFinder/ (accessed on 18 February 2018)). Prophage regions were identified using the RAST and Phaster resources [197,198]. Additional analysis of pathogenicity factor genes, as well as their products, was performed manually on the basis of the NCBI BLASTp platform and using the Clustal resource.

## 5. Conclusions

*S. aureus* acquired numerous determinants of pathogenicity, evolving together with humans for a long time. This allows the microbe to colonize many epitopes, resist the immune system of the host, and cause various diseases. Pathogen proteins are multifunctional; they have many ligands, as a rule, and take part in various patterns of pathogen–host interaction. The multiple duplication of proteins that perform key pathogenetic tasks (functions) is one of the main strategies developed by *S. aureus*, which is clearly reflected in its genome. The pathogen has formed several multicomponent and mutually subordinate systems for regulating the expression of virulence, providing it with perfect mechanisms for adaptation and survival, both as planktonic cells and in the form of a biofilm. Fine regulation of the expression of pathogenicity factors and their cooperation at various stages of infection allow the pathogen to maintain the activity and duration of the inflammatory process in various environments and host tissues. A unique feature of the pathogen is its ability to sacrifice a part of the mobile genetic pool in order to more effectively tune the work of the pathogenicity gene repertoire and acquire additional advantages in solving urgent problems of confrontation with the macroorganism. On experimental models and as a result of the analysis of clinical observations, it has been ascertained that the appearance of mutations in key pathogenicity genes or the loss of one or even several pathogenicity factors does not always unequivocally lead to a decrease in the virulence of the pathogen. The rapid formation of mechanisms of resistance to antimicrobial drugs significantly limits the chances of effective antibacterial therapy for staphylococcal diseases. It is becoming increasingly clear that strategies for both the prevention and treatment of staphylococcal infections must be simultaneously targeted at multiple pathogen molecular targets, and the drugs created should be adapted to different types of infectious process. In this regard, achieving real success in the development of antistaphylococcal drugs aimed at suppressing one or even several pathogenicity factors of *S. aureus* seems to be an extremely difficult task. Our findings provide a platform for future investigation using virtual screening and molecular docking to predict molecular targets and development drugs aimed at neutralizing pathogenicity determinants that prevent the formation of mature immunological memory. This would allow the host to form an effective immune response. An alternative approach may be to search for and block molecular targets in the common metabolic pathways or in peptidoglycan biosynthesis of this universal pathogen.

## Figures and Tables

**Table 1:** *Staphylococcus aureus* strains.

Strain	Accession Number of the Genbank NCBI	Genetic Features			Country	Year
		Sensitivity to Oxacillin	MLST Data	Belonging to Subclones ^#^		
NCTC 8325	LS483365.1	MSSA *	8	-	UK	before 1949
Newman	AP009351.1	MSSA	8	-	UK	1952
Col	CP000046.1	MSSA	250	-	UK	1961
FPR3757	CP000255.1	MRSA **	8	-	USA	before 2002
N315	NC_002745.2	HA-MRSA	5	-	Japan	1982
MW2	NC_003923.1	CA-MRSA	1	-	USA	1998
MRSA252	BX571856.1	HA-MRSA	36	-	UK	1997
55/2053	CP002388.1	HA-MRSA	30	-	USA	2009
T0131	CP002643.1	HA-MRSA	239	1	China	2006
TW20	FN433596.1	HA-MRSA	239	2	UK	2003
Z172	CP006838.1	HA-MRSA	239	2	Taiwan	2010
V521	CP013957.1	HA-MRSA	239	2	South Korea	2011
Bmb9393	CP005288.1	HA-MRSA	239	3	Brazil	1993
Be62	CP012013.1	HA-MRSA	239	3	Brazil	1996
JKD6008	CP002120.1	HA-VISA ***	239	4	New Zealand	2003

Note: *—methicillin-sensitive; **—methicillin-resistant; ***—vancomycin-resistant. ^#^: 1—Eurasian subclone; 2—South Asian subclone; 3—American/-Middle Eastern subclone; 4—Australian/New Zealand sub-clone.

## Data Availability

The results of whole genome sequencing of *S. aureus* 943 are presented in DDBJ/ENA/GenBank under number QLNS00000000, as well as in the SRA NCBI read archive (accession PRJNA476233). It was written in Section 2.1 Additionally: Submission data: 24 June 2018.

## Supplementary Materials

The following supporting information can be downloaded at: https://www.mdpi.com/article/10.3390/ijms232416086/s1.
