# Supplementary material for: In Silico Genome-Scale Analysis of Molecular Mechanisms Contributing to the Development of a Persistent Infection with Methicillin-Resistant Staphylococcus aureus (MRSA) ST239
Source: Int J Mol Sci. 2022 Dec 16;23(24):16086. doi: 10.3390/ijms232416086 (PMC9781258; doi:10.3390/ijms232416086)
Supplement: Supplementary file 1 [file ijms-23-16086-s001.zip › ijms-2033084-supplementary.pdf]

Supplementary Table S1. *S. aureus* SA943 virulom.

Protein group	Protein	Protein accesssion number
1	2	3
**Hemolysins**	Alpha-hemolysin	RAM45765.1
	Beta-hemolysin	RAM45197.1
	Gamma-hemolysin subunit A	RAM47209.1
	Gamma-hemolysin subunit B	RAM47121.1
	Gamma-hemolysin subunit C	RAM47120.1
	Hemolysin III	RAM45352.1
**Leukocidins**	LukED subunit D	RAM45663.1
	LukED subunit E	RAM45662.1
	LukGH subunit H	RAM45195.1
	LukGH subunit G	RAM45196.1
**Phenol-soluble modulins**	Phenol-soluble modulin export ABC transporter ATP-binding protein PmtA	RAM45801.1
	Phenol-soluble modulin export ABC transporter permease subunit PmtB	RAM45802.1
	Phenol-soluble modulin export ABC transporter ATP-binding protein PmtC	RAM45803.1
	Phenol-soluble modulin export ABC transporter permease subunit PmtD	RAM45804.1
	Beta-class phenol-soluble modulin	RAM45753.1
	Beta-class phenol-soluble modulin	RAM45754.1
	Phenol-soluble modulin PSM-alpha-1	RAM45730.1
	Phenol-soluble modulin PSM-alpha-2	RAM45731.1
	Phenol-soluble modulin PSM-alpha-3	RAM45732.1
	Phenol-soluble modulin PSM-alpha-4	RAM45733.1
	Delta-lysin family phenol-soluble modulin	RAM45554.1
	Phenol-soluble modulin PSM-mec	RAM45233.1
**Enterotoxins**	Enterotoxin Q (SEQ)	RAM45503.1
	Enterotoxin K (SEK)	RAM45504.1
	Enterotoxin A	RAM45425.1
	Enterotoxin 26	RAM47603.1
**Superantigen-lilke proteins(SSL)**	SSL1	RAM45984.1
	SSL2	RAM45983.1
	SSL3	RAM45982.1
	SSL4	RAM45980.1
	SSL5	RAM45979.1
	SSL6	RAM45978.1
	SSL7	RAM45977.1
	SSL8	RAM45976.1
	SSL9	RAM45975.1
	SSL10	RAM45974.1
	SSL11	RAM45728.1
	SSL12	RAM45762.1
	SSL13	RAM45761.1
	SSL14	RAM45760.1
**Serine (Spl) and cysteine proteases**	Serine protease (SplA)	RAM45676.1
	Serine protease (SplB)	RAM45677.1
	Serine protease (SplC)	RAM45678.1
	Serine protease (SplF)	RAM45679.1
	Мембраносвязанная сериновая протеаза	RAM47657.1
	Serine endopeptidase SspA	RAM47318.1
	S41 family peptidase	RAM47419.1
	Rhomboid family intramembrane serine protease	RAM47553.1
	Cysteine protease staphopain B	RAM47317.1
	Cysteine protease staphopain A	RAM45827.1

Supplementary Table S1 (continued).

1	2	3
**Serine (Spl) and cysteine proteases**	Staphostatin B	RAM47316.1
	S8 family serine peptidase	RAM45670.1
	Zinc metalloproteinase aureolysin	RAM45971.1
**Adhesions and invasions**	Coagulase	RAM47813.1
	Catalase	RAM46449.1
	Thermonuclease	RAM46449.1
	YSIRK domain-containing triacylglycerol Lip1	RAM45969.1
	Glycerol kinase GlpK	RAM46556.1
	Aerobicglycerol-3-phosphate dehydrogenase	RAM46555.1
	Immunoglobulin G-binding protein A	RAM47916.1
	Immunoglobulin G-binding protein Sbi	RAM47117.1
	MSCRAMM family adhesin clumping factor ClfA	RAM45532.1
	MSCRAMM family adhesin clumping factor ClfB	RAM45959.1
	Cna B-type domain-containing protein	RAM46073.1
	Elastin-binding protein EbpS	RAM47482.1
	Extracellular matrix protein-binding adhesin Emp	RAM45531.1
	MSCRAMM family adhesin SdrE	RAM46170.1
	MSCRAMM family adhesin SdrC	RAM46168.1
	MSCRAMM family adhesin SdrD	RAM46169.1
	Serine-enriched repetitive glycoprotein adhesin SasA	RAM45936.1
	Cell wall anchored protein SasF	RAM45943.1
	Virulence-assoiated cell-wall anchored protein SasG	RAM47201.1
	Class A sortase SrtA	RAM46780.1
	Class B sortase SrtB	RAM45126.1
	Fibronectin-binding protein A	RAM46647.1
	Complement inhibitor SCIN-B	RAM45768.1
	Efb fibrinogen-binding protein	RAM45769.1
	Ecb complement convertase inhibitor	RAM45773.1
	Intercellular adhesin biosynthesis polysaccharide N-deacetylase	RAM45922.1
	Polysaccharide biosynthesis protein	RAM45388.1
	Poly-beta-1,6-N-acetyl-D-glucosamine synthesis protein IcaD	RAM45923.1
	Poly-beta-1,6 N-acetyl-D-glucosamine synthase IcaA	RAM45924.1
	Ica operon transcriptional regulator IcaR	RAM45925.1
	Capsule biosynthesis protein CapA	RAM45926.1
	Polysaccharide biosynthesis tyrosine autokinase	RAM45927.1
	Tyrosine-protein phosphatase	RAM45928.1
	Type 8 capsular polysaccharide synthesis protein Cap8K	RAM47868.1
	Type 8 capsular polysaccharide synthesis protein Cap8I	RAM47870.1
	Type 8 capsular polysaccharide synthesis protein Cap8H	RAM47871.1
	Type 8 capsular polysaccharide synthesis protein Cap8F	RAM47873.1
	Type 8 capsular polysaccharide synthesis protein Cap8C	RAM47876.1
	Type 8 capsular polysaccharide synthesis protein Cap8B	RAM47877.1
	LPXTG-anchored adenosine synthase AdsA	RAM46119.1
	BsaG protein	RAM45673.1
	Extracellular adherence protein Eap/Map	RAM45198.1
	MAP domain-containing protein	RAM45199.1
**Regulatory factors**	Accessory gene regulator protein A AgrA	RAM45557.1
	Accessory gene regulator protein B AgrB	RAM45555.1
	Вспомогательный генный регулятор AgrC	RAM45580.1
	Cyclic lactone autoinducer peptide (regulator protein agrD)	RAM45556.1
	Global transcriptional regulator SarA	RAM46821.1
	HTP- type transcriptional regulator SarR	RAM46294.1
	HTP- type transcriptional regulator SarS	RAM47915.1
	HTP- type transcriptional regulator SarT	RAM47202.1
	HTP-type transcriptional regulator SarX	RAM47004.1
	Регулятор транскрипции SarZ	?

Supplementary Table S1 (continued).

1	2	3
**Regulatory factors**	HTP- type transcriptional regulator SarV	RAM46266.1
	HTP- type transcriptional regulator SarU	RAM46810.1
	HTP- type transcriptional regulator MgrA	RAM46890.1
	HTP-type transcriptional regulator| MarR(Rot) (Repressor of toxins Rot)	RAM45395.1
	Metal-dependent transcriptional regulator|	RAM46837.1
	AraC family transcriptional regulator|	RAM46871.1
	LysR family transcriptional regulator	RAM46876.1
	TetR/AcrR family transcriptional regulator	RAM46695.1
	TetR/AcrR family transcriptional regulator	RAM46731.1
	Sugar-binding transcriptional regulator DeoR	RAM46982.1
	Helix-turn-helix transcriptional regulator	RAM46996.1
	RNA polymerase sigma factor Sig B	RAM46417.1
	Anti-Sig B factor RsbW	RAM46416.1

Supplementary Table S2a. *Staphylococcus aureus* protein A (Spa) amino acid sequence: amino acid substitution localization.

Supplementary Table S2a. *Staphylococcus aureus* protein A (Spa) amino acid sequence: amino acid substitution localization.

Strain	Protein accession number	Aa number	Query Cover (%)	Per. Ident (%)	Amino acid substitutions									
					Protein region									
					S	D			A	C				
					13	99	104	120	199	292	332	339	419	461
NCTC8325-4	AAA26676.1	524	100	100	M	N	Q	A	S	T	N	N	G	D
NCTC 8325	SQF72440.1	516	100	98,47	M	N	Q	A	S	T	N	N	G	D
Newman	BAF66327.1	520	97	96,76	***L***	N	Q	A	S	T	N	N	G	D
Col	AAW38738.1	508	100	96,56	M	N	Q	A	S	T	N	N	G	D
FPR3757	ABD22331.1	508	100	96,56	M	N	Q	A	S	T	N	N	G	D
N315	BAB41326.1	492	100	82,25	M	N	Q	A	S	***N***	***K***	***G***	G	D
MRSA252	CAG39140.1	516	100	96,97	M	***K***	Q	***E***	***A***	T	N	N	G	D
MW2	BAB93949.1	450	100	92,94	M	N	Q	A	S	T	N	***G***	***N***	D
55/2053	EEV05371.2	442	100	79,01	M	***K***	Q	***E***	***A***	T	N	N	G	D
Bmb9393	AGP27111.1	418	83	83,33	M	***K***	Q	***E***	***A***	T	N	N	G	D
Be62	ALY21933.1	438	100	75,76	***L***	***K***	Q	***E***	***A***	T	N	N	G	D

TW20	CBI48007.1	426	100	75,95	M	***K***	Q	***E***	***A***	T	N	N	G	D
JKD6008	ADL64170.1	438	100	75,57	***L***	***K***	***R***	***E***	***A***	T	N	N	G	D
T0131	WP_000728717.1	418	100	82,42	M	***K***	***R***	***E***	***A***	T	N	N	G	***A***
Z172	AGY88223.1	426	100	75,95	M	***K***	Q	***E***	***A***	T	N	N	G	D
V521	WP_000728706.1	426	100	75,95	M	***K***	Q	***E***	***A***	T	N	N	G	D
SA943	RAM47916.1	418	100	82,42	M	***K***	***R***	***E***	***A***	T	N	N	G	***A***

Note: domain localization is indicated in strain NCTC 8325-4 according to (Uhlen M, 1984): 1-39 aa - signal sequence (S); 40-93 aa – E domain; 94-150 aa - D domain; 156-213 aa - A domain; 214-274 aa - B domain; 275-342 aa - C domain; 339-441 aa - X-variable region; 469-524 aa - C-terminus, including LPxTG motif (490-494 aa) peptidoglycan attachment region.

Table S2b. *Staphylococcus aureus* protein A (Spa) amino acid sequence: localization of insertions and deletions.

Strain	Protein accession number	Insertions	Deletions												
		S	Domains EDBAC (IgG binding region)		X - region										
		1-12	**130-187**	226-283	344-362	336-343	344-375	354-369	357-372	**360-407**	380-395	388-395	397-409	393-409	398-405
NCTC8325-4	AAA26676.1	-	-	-	-	-	-	-	-	-	-	-	-	-	-
NCTC8325	WP_000728764.1	-	-	-	-	+	-	-	-	-	-	-	-	-	-
Newman	BAF66327.1	+	-	-	-	-	-	+	-	-	-	-	-	-	-
Col	AAW38738.1	-	-	-	-	-	-	-	-	-	-	+	+	-	-
FPR3757	ABD22331.1	-	-	-	-	-	-	-	-	-	-	-	-	+	-
N315	BAB41326.1	-	-	-	-	-	-	-	+	-	-	-	-	-	-
MRSA252	CAG39140.1	-	-	-	-	-	-	-	-	-	-	-	-	-	-
MW2	BAB93949.1	-	-	+	-	-	+	-	-	-	-	-	-	-	-
55/2053	EEV05371.2	-	+	-	-	-	-	-	-	-	+	-	-	-	+
Bmb9393	AGP27111.1	-	+	-	+	-	-	-	-	-	+	-	-	-	+

Be62	ALY21933.1	+	+	-	-	-	-	+	-	-	+	-	-	-	+
TW20	CBI48007.1	-	+	-	-	-	-	+	-	-	+	-	-	-	+
JKD6008	ADL64170.1	+	+	-	-	-	-	+	-	-	+	-	-	-	+
T0131	WP_000728717.1	-	+	-	-	-	-	-	-	+	-	-	-	-	-
Z172	AGY88223.1	-	+	-	-	-	-	+	-	-	+	-	-	-	+
V521	WP_000728706.1	-	+	-	-	-	-	+	-	-	+	-	-	-	+
SA943	RAM47916.1	-	+	-	-	-	-	-	-	+	-	-	-	-	-

Note: The presence of deletions is marked with a cross on a light gray background.

Supplementary Table S3a. S*taphylococcus aureus* fibronectin-binding protein A (FnBPA) amino acid sequence: localization of amino acid substitutions.

Strain	Protein accession number	Number of amino acids	Query Cover (%)	Per Ident (%)	Localization and number of substitutions in different protein regions						Total number of substitutions
					S	Nv	Fg	Fn	C PPR	C (WMC)	
					1-37	38-195	194-511	512-885	886-993	994-1079	
NCTC8325	WP_000794582.1	**1018**	100	100	-	-	-	-	-	-	0
Newman	BAF68671.1	741	100	99,19	-	-	***E509G***	***+ (4)***	-	-	***5***
Col	WP_000794589.1	**1018**	100	99,8	-	-	***E509G***	***K703E***	-	-	***2***
FPR3757	WP_000794589.1	**1018**	100	99,8	-	-	***E509G***	***K703E***	-	-	***2***
N315	WP_000794614.1	1038	83	83,28	-	***+ (13)***	+ ***(98)***	***+ (28)***	-	-	***139***
MRSA252	WP_000794580.1	965	86	78,38	+ ***(1)***	***+ (11)***	***+ (106)***	***+ (32)***	-	-	***150***
MW2	WP_000794648.1	1015	85	84,08	-	***+ (12)***	***+ (109)***	***+ (28)***	-	-	***149***
55/2053	WP_000794642.1	1042	83	82,27	-	***+ (11)***	***+ (104)***	***+ (38)***	-	-	***153***

Bmb9393	AGP29431.1	**1018**	100	99,8	-	-	***E509G***	***K703E***	-	-	***2***
Be62	WP_000794589.1	**1018**	100	99,8	-	-	***E509G***	***K703E***	-	-	***2***
TW20	CBI50487.1	995	97	99,8	-	-	***E509G***	***K703E***	-	-	***2***
JKD6008	WP_000794589.1	**1018**	100	99,8	-	-	***E509G***	***K703E***	-	-	***2***
T0131	WP_000794592.1	990	100	96,56	-	-	***E509G***	***K703E***	+ (*4*)[1]	-	*7*
Z172	AGY90698.1	967	100	96,98	-	-	***E509G***	***K703E***	-	-	***2***
V521	WP_000794589.1	**1018**	100	99,8	-	-	***E509G***	***K703E***	-	-	***2***
SA943	RAM46808.1	990	100	96,56	-	-	***E509G***	***K703E***	+ (*4*)[1]	-	*7*

Note: S - signal sequence; Nv-N-terminus, variable region; Fg-fibrinogen and elastin-binding domain A; Fn - fibronectin-binding domain; PPR, proline-rich repeat region; C - C-terminus, including cell wall (W), membrane (M) and cytosolic region (C) attachment domains; [1] - amino acid substitutions: ***V936I; A970V; P975A; K993N.***

Supplementary Table S3b. S*taphylococcus aureus* fibronectin-binding protein A (FnBPA): amino acid sequence: localization of deletions.

Strain	Protein accession number	Protein regions							
		S	Nv	Fn				C 886-993	
		1-23	115-119	763-800	876-878	879-902	880-918	898-925[1]	892-920
NCTC 8325	WP_000794582.1	-	-	-	-	+	-	-	-
Newman	BAF68671.1	-	-	-	-	-	-	-	-
Col	WP_000794589.1	-	-	-	-	+	-	-	-
FPR3757	WP_000794589.1	-	-	-	-	+	-	-	-
N315	WP_000794614.1	-	-	-	-	-	-	-	-
MRSA252	CAG41560.1	-	-	+	+	-	+	-	-
MW2	WP_000794648.1	-	+	-	-	-	-	+	-
55/2053	WP_000794642.1	-	-	-	+	-	-	-	-
Bmb9393	AGP29431.1	-	-	-	-	+	-	-	-
Be62	WP_000794589.1	-	-	-	-	+	-	-	-
TW20	CBI50487.1	+	-	-	-	+	-	-	-

JKD6008	WP_000794589.1	-	-	-	-	+	-	-	-
T0131	WP_000794592.1	-	-	-	-	+	-	-	+
Z172	AGY90698.1	+	-	-	-	+	-	-	+
V521	WP_000794589.1	-	-	-	-	+	-	-	-
SA943	RAM46808.1	-	-	-	-	+	-	-	+

Note: - deletions are localized at the end of the fibronectin-binding region and capture the repeating region; [1] - the localization of this deletion in WP_000794648[1] of *S. aureus* MW2 is indicated when it was aligned with WP_000794614.1. The presence of deletions was marked with a cross on a light gray background.

Supplementary Table S4. *Staphylococcus aureus* SdrD amino acid sequence: localization of amino acid substitutions and deletions.

Strain	Protein accession number	Number of amino acids	Query Cover (%)	Per. Ident (%)	Number of amino acid substitutions in the structural domains of the protein						Number of SD dipeptides
					S 1-35	A 36-550	Spacer region 551-559	Subdomains B (B1-B5) 560-1113	R 1114-1320	WMC 1314-1374	
1	2	3	4	5	6	7	8	9	10	11	12
NCTC 8325	SQF72859.1	1349	100	**97.32**	-	-	-	3	1	2	80
Newman	WP_000934419.1	1315	**95**	**99.92**	-	-	-	-	1	-	63
Col	WP_000934424.1	1381	100	100	-	-	-	-	-	-	96
FPR3757	WP_000934424.1	1381	100	100	-	-	-	-	-	-	96
N315	WP_000934467.1	1385	**81**	**93.77**	-	62	-	2	1	-	98
MW2	WP_000934435.1	1347	100	**96.16**	-	13	-	3	1	-	79
55/2053	AGT63464.1	1360	**81**	**82.77**	-	151	-	17	-	-	93
Bmb9393	AGP27562.1	1381	100	100	-	-	-	-	-	-	96
Be62	WP_000934424.1	1381	100	100	-	-	-	-	-	-	96

TW20	CBI48511.1	1381	100	**99.93**	-	1	-	1		-	96

Supplementary Table S4. *Staphylococcus aureus* SdrD amino acid sequence: localization of amino acid substitutions and deletions (continued).

1	2	3	4	5	6	7	8	9	10	11	12
JKD6008	WP_000934423.1	1375	100	**97.57**	-	-	-	-	-	-	93
T0131	WP_000934424.1	1381	100	100	-	-	-	-	-	-	96
Z172	AGY88666.1	1369	100	**98.99**	-	-	-	1	1	-	90
V521	WP_063655687.1	1375	100	**99.42**	-	1	-	1	1	-	93
SA943	RAM46169.1	1381	100	100	-	-	-	-	-	-	96
MRSA252	No protein	-	-	-	-	-	-	-	-	-	-

Note: S - N- terminal signal sequence; A - domain with ligand binding N2/N3 subdomains; B1-B5 - recurring B subdomains; R - serine-aspartate dipeptide region; WMC - wall-spanning segment.

Supplementary Table S5. *Staphylococcus aureus* SdrE amino acid sequence: localization of amino acid substitutions and deletions.

Strain	Protein accession number	Aa number	Query cover (%)	Per. ident (%)	Amino acid substitutions													
					Protein region													
					A													
					78	87	106	110	115	160	227	240	244	261	285	385	396	397
Col	AAW37719.1	1166	100	100	T	N	K	S	K	N	***K***	S	H	V	K	S	K	T
Newman	BAF66797.1	1166	100	**99.91**	T	N	K	S	K	N	***N***	S	H	V	K	S	K	T
FPR3757	ABD22410.1	1154	100	**98.80**	T	N	K	S	K	N	***N***	S	H	V	K	S	K	T
N315[1]	BAB41752.1	1141	100	**97.72**	T	***D***	***E***	***T***	***Q***	***Y***	***N***	***N***	***R***	***L***	***T***	***A***	***Q***	***A***
MRSA252[1]	CAG39588.1	1137	80	**83.94**	T	***D***	***E***	***P***	***Q***	N	***N***	S	***R***	V	***T***	***A***	K	***E***
MW2[1]	BAB94383.1	1141	100	**98.95**	***A***	***D***	***E***	***K***	***Q***	N	***N***	S	H	V	K	S	K	T
55/2053	No protein	-	-	**-**	-	-	-	-	-	-	-	-	-	-	-	-	-	-
Bmb9393	AGP27563.1	1161	100	**99.40**	T	N	K	S	K	N	***N***	S	H	V	K	S	K	T
Be62	ALY22396.1	1161	100	**99.40**	T	N	K	S	K	N	***N***	S	H	V	K	S	K	T
TW20	CBI48512.1	1137	100	**97.34**	T	N	K	S	K	N	***N***	S	H	V	K	S	K	T
JKD6008	ADL64631.1	1133	100	**97.00**	T	N	K	S	K	N	***N***	S	H	V	K	S	K	T

T0131	AEB87697.1	1131	100	**96.83**	T	N	K	S	K	N	***N***	S	H	V	K	S	K	T
Z172	n/d	-	-	**-**	-	-	-	-	-	-	-	-	-	-	-	-	-	-
V521	AND35210.1	1137	100	**97.34**	T	N	K	S	K	N	***N***	S	H	V	K	S	K	T
SA943	RAM46170.1	1029	100	**99.18**	T	N	K	S	K	N	***N***	S	H	V	K	S	K	T

Supplementary Table S5. *Staphylococcus aureus* SdrE amino acid sequence: localization of amino acid substitutions and deletions (continued).

Strain	Protein accession number	Amino acid substitutions													Deletions / localization		
		Protein region													Protein region		
		A						B1 606-719			B2 720-829		B3 830-939		A	R	C
		420	444	460	501	502	594	655	677	699	800	823	858	862	174-178[3]	number of SD repeats/ (-number of deletions)	
Col	AAW37719.1	T	D	S	S	D	T	N	T	V	D	R	T	Q	-	83/0	-
Newman	BAF66797.1	T	D	S	S	D	T	N	T	V	D	R	T	Q	-	83/0	-
FPR3757	ABD22410.1	T	D	S	S	D	T	N	T	V	D	**S**	T	Q	-	77/ **(-6)**	-
N315[1]	BAB41752.1	***S***	***N***	***T***	***P***	***N***	***S***	N	***A***	***I***	***E***	**S**	***K***	***L***	+	73/ **(-10)**	-
MRSA252[1]	CAG39588.1	***K***	***T***	***N***	S	***G***	***S***	N	***A***	V	***E***	***S***	T	Q	+	71/ **(-12)**	-
MW2[1]	BAB94383.1	T	D	S	S	D	T	***K***	T	V	***E***	**S**	T	Q	+	73/ **(-10)**	-
55/2053	No protein	-	-	-	-	-	-	-	-	-	-	-	-	-	-	-	-
Bmb 9393	AGP27563.1	T	D	S	S	D	T	N	T	V	D	**S**	T	Q	+	83 /(0)	-
Be62	ALY22396.1	T	D	S	S	D	T	N	T	V	D	**S**	T	Q	+	83 / **(0)**	-
TW20	CBI48512.1	T	D	S	S	D	T	N	T	V	D	R	T	Q	+	71/ **(-12)**	-
JKD6008	ADL64631.1	T	D	S	S	D	T	N	T	V	D	**S**	T	Q	+	69/ **(-14)**	-
T0131	AEB87697.1	T	D	S	S	D	T	N	T	V	D	**S**	T	Q	+	68/ **(-15)**	-
Z172	n/d	?	?	?	?	?	?	?	?	?	?	?	?	?	**?**	?	?
V521	AND35210.1	T	D	S	S	D	T	N	T	V	D	**S**	T	Q	+	71/ **(-12)**	**18[2]**
SA943	RAM46170.1	T	D	S	S	D	T	N	T	V	D	**S**	T	Q	+	17/ **(-66)**	-

Note: The SdrE regions: ligand-binding domain A; subdomains B1-3(function is unknown); R-region, containing Ser-Asp dipeptides; C-terminal end, that is involved in the protein attachment to the bacterial cell wall; [1] - bone sialoprotein-binding protein, that is allelic variant of SdrE; *S. aureus* MRSA252 carries more than 60 amino acid substitution additionally; [2]- the number of amino acids that have been lost; [3]- deletions of 5 amino acids: TSEPS.

Supplementary Table S6. *Staphylococcus aureus* hemolysins/leucocidins amino acid sequence.

Strain	Toxin/ number of amino acids (aa)			Toxin/ number of amino acids (aa)			Toxin/ number of amino acids (aa)		
	Hla /319 aa			LukED subunit E/311aa			LukED subunit D/327 aa		
	Protein accession number	Per. Ident (%)	Aa sub-stitutions	Protein accession number	Per. Ident (%)	Aa sub-stitutions	Protein accession number	Per. Ident (%)	Aa sub-stitutions
NCTC 8325	WP_000857483.1	100	no	WP_000473596.1	100	no	WP_000782464.1	100	no
Newman	WP_000857483.1	100	no	WP_000473596.1	100	no	WP_000782464.1	100	no
Col	WP_000857483.1	100	no	WP_000473596.1	100	no	WP_000782464.1	100	no
FPR3757	WP_000857483.1	100	no	WP_000473596.1	100	no	WP_000782464.1	100	no
N315	WP_000857488.1	***99.37***	**D234E; I301T**	WP_000473596.1	100	no	WP_000782463.1	***99.69***	**V287T**
MW2	WP_000857485.1	***99.69***	**I301T**	WP_000473596.1	100	no	WP_000782464.1	100	no
MRSA252	CAG40139.1[1]	**-**	**-**	n/d	n/d	n/d	n/d	n/d	n/d
55/2053	EEV04322.1	***98.43***	**78 I/L; I301T**	n/d	n/d	n/d	n/d	n/d	n/d
TW20	WP_000857484.1	***99.69***	**R4T**	WP_000473590.1	***99,68***	G154A	WP_000782464.1	100	no
T0131	WP_000857484.1	***99.69***	**R4T**	WP_000473596.1	100	no	AEB88916.1[2]	**85**/100	

Z172	WP_000857484.1	***99.69***	**R4T**	WP_000473590.1	***99,68***	G154A	WP_000782464.1	100	no
V521	WP_000857485.1	***99.68***	**R4T**	AND36441.1	***99,68***	G154A	AND36440.1	100	no
Bmb9393	WP_000857484.1	***99.69***	**R4T**	WP_000473596.1	100	no	WP_000782464.1	100	no
Be62	WP_000857484.1	***99.69***	**R4T**	ALY23745.1	***100***	no	ALY23744.1	100	no
JKD6008	WP_000857484.1	***99.69***	**R4T**	?	***?***	?	?	?	?
943	RAM45765.1	***99.69***	**R4T**	RAM45662.1	100	no	RAM45663.1	100	no

Supplementary Table S6. *Staphylococcus aureus* hemolysins/leucocidins amino acid sequence (continued).

Strain	Toxin/ number of amino acids (aa)			Toxin/ number of amino acids (aa)			Toxin/ number of amino acids (aa)		
	Gamma-hemolysin subunit A/309 aa			Gamma-hemolysin subunit B/325 aa			Gamma-hemolysin C/319 aa		
	Protein accession number	Per. Ident (%)	Aa sub-stitutions	Protein accession number	Per. Ident (%)	Aa substitutions	Protein accession number	Per. Ident (%)	Aa sub-stitutions
NCTC 8325	WP_000594519.1	100	no	WP_000783428.1	100	no	WP_000916713.1	100	no
Newman	WP_000594519.1	100	no	WP_000783428.1	100	no	WP_000916713.1	100	no
Col	WP_000594519.1	100	no	WP_000783428.1	100	no	?		no
FPR3757	WP_000594519.1	100	no	WP_000783428.1	100	no	WP_000916713.1	100	no
N315	WP_000594519.1	100	no	WP_000783428.1	100	no	WP_000916704.1	**99.68**	***T8A***
MW2	WP_000594519.1	100	no	WP_000783428.1	100	no	WP_000916704.1	**99.68**	***T8A***
MRSA252	WP_000594517.1	**99.35**	***R246K; A264T***	WP_000783428.1	100	no	CAG41490.1	**97.78**	***T8A***[4]

55/2053	EEV05022.1[3]	**99.68**	***E241K***	WP_001056917.1	**97.54**	***K2N; G22N; T82A; F89Y; V90E; K91R; N95K; L102T***	EEV05023.1	**97.78**	***T8A***[4]
TW20	WP_000594519.1	100	no	WP_000783428.1	100	no	WP_00096713.1	100	no
To131	WP_000594519.1	100	no	WP_000783428.1	100	no	WP_00096713.1	100	no
Z172	WP_000594519.1	100	no	WP_000783428.1	100	no	WP_00096713.1	100	no
V521	AND37622.1	100	no	AND37245.1	100	no	AND37244.1	100	no
Bmb9393	WP_000594519.1	100	no	WP_000783428.1	100	no	WP_00096713.1	100	no
Be62	ALY24329.1[3]	100	no	ALY24332.1	100	no	ALY24331.1	100	no
JKD6008	WP_000594519.1	100	no	WP_000783428.1	100	no	WP_00096713.1	100	no
943	RAM47209.1	100	no	RAM47121.1	100	no	RAM47120.1	100	no

Supplementary Table S6. *Staphylococcus aureus* hemolysins/leucocidins amino acid sequence (continued).

Strain	Toxin/ number of amino acid (aa)			Toxin/ number of amino acid (aa)			Toxin/ number of amino acid (aa)		
	LukGH subunit G/338aa			LukGH subunit H/ 351aa			Hemolysin III /227aa		
	Protein accession number	Per. Ident (%)	Aa substitutions	Protein accession number	Per. Ident (%)	Aa substitutions	Protein accession number	Per. Ident (%)	Aa substitu-tions
NCTC 8325	WP_000595324.1	100	no	WP_000791407.1	100	no	WP_000047255.1	100	no
Newman	WP_000595324.1	100	no	WP_000791407.1	100	no	WP_000047255.1	100	no
Col	WP_000595324.1	100	no	WP_000791407.1	100	no	WP_000047255.1	100	no
FPR3757	WP_000595324.1	100	no	WP_000791407.1	100	no	WP_000047255.1	100	no
N315	WP_000595392.1	**97.34**[5]	**8**	WP_000791410.1	100	no	WP_000047255.1	100	no
MW2	n/d	n/d	n/d	n/d	n/d	n/d	WP_000047255.1	100	no
MRSA252	n/d	n/d	n/d	n/d	n/d	n/d	WP_000047120.1	**98.68**	***T4S; E5K***
55/2053	n/d	n/d	n/d	n/d	n/d	n/d	WP_000047120.1	**98.68**	***T4S; E5K***
TW20	WP_000595324.1	100	no	WP_000791407.1	100	no	WP_000047255.1	100	no
To131	WP_000595324.1	100	no	WP_000791407.1	100	no	WP_000047255.1	100	no

Z172	WP_000595324.1	100	no	WP_000791407.1	100	no	WP_000047255.1	100	no
V521	WP_000595324.1	100	no	AND36691.1	100	no	AND36982.1	100	no
Bmb9393	n/d	n/d	n/d	WP_000791407.1	100	no	WP_000047255.1	100	no
Be62	ALY23926.1	100	no	ALY2396.7	100	no	ALY24076.1	100	no
JKD6008	WP_000595323.1	**99.7**	***K308E***	WP_000791407.1		no	WP_000047255.1	100	no
943	RAM45196.1	**100**	no	RAM45195.1	100	no	RAM45352.1	**99.56**	***I96T***

Note: [1]- pseudogene due to the presence of a stop codon at position 112; [2]- length 281 aa as a result of deletion of a fragment of 46 aa, starting from aa 282; [3]- length 321 aa as a result of insertion 12 aa at the beginning of the sequence; [4]- amino acid substitutions were identified additionally: ***S40N*****;** ***I42V; V92K; K96N; V100I; A102S*****;** [5] - amino acid substitutions were identified**:** ***C6Y; T13S; L16I; T19A; F20L; F23Y; Q32A; N325D.***

Supplementary Table S7. *Staphylococcus aureus* PSM peptides amino acid sequence.

Strain	Peptide accession number / number of amino acids (aa)					
	Alpha-1/21aa	Alpha-2/21aa	Alpha-3/22aa	Alpha-4/20aa	Beta-1/44aa	Beta-2/44aa
NCTC 8325	P0C7Y1.1	P0C7Z3.1	P0C805.1	n/d	WP_000147103.1	Wp_000398672.1
Newman	WP_014373781.1	WP_014373780.1	WP_014373779.1	WP_014532416.1	WP_000147103.1	Wp_000398672.1
Col	WP_014373781.1	WP_014373780.1	WP_014373779.1	WP_014532416.1	WP_000147103.1	Wp_000398672.1
FPR3757	WP_014373781.1	WP_014373780.1	WP_014373779.1	WP_014532416.1	WP_000147103.1	Wp_000398672.1
N315	WP_014373781.1	WP_014373780.1	WP_014373779.1	WP_014532416.1	WP_000147103.1	Wp_000398672.1
MW2	WP_014373781.1	WP_014373780.1	WP_014373779.1	WP_014532416.1	WP_000147103.1	Wp_000398672.1
MRSA252	Wp_014373781.1	WP_014373780.1	WP_099119694.1	WP_014532416.1	n/d	Wp_000398672.1
T0131	WP_014373781.1	WP_014373780.1	WP_014373779.1	WP_014532416.1	WP_000147103.1	Wp_000398672.1
55/2053	WP_014373781.1	WP_014373780.1	n/d	n/d	n/d	n/d
TW20	WP_014373781.1	WP_014373780.1	WP_014373779.1	WP_014532416.1	WP_000147103.1	Wp_000398672.1
Z172	WP_014373781.1	WP_014373780.1	WP_014373779.1	WP_014532416.1	WP_000147103.1	n/d
V521	n/d	n/d	n/d	n/d	n/d	n/d
Bmb9393	WP_014373781.1	WP_014373780.1	WP_014373779.1	n/d	WP_000147103.1	Wp_000398672.1
Be62	n/d	n/d	n/d	n/d	n/d	n/d
JKD6008	WP_014373781.1	Wp_014373780.1	Wp_14373779.1	Wp_014532416.1	Wp_000147103.1	Wp_000398672.1
SA943	RAM45730.1	RAM45731.1	RAM45732.1	RAM45733.1	RAM45753.1	RAM45754.1

Supplementary Table S7. *Staphylococcus aureus* PSM peptides amino acid sequence (continued).

Strain	Peptide accession number / number of amino acids (aa)					
	Delta-lysin/44aa	PSM*mec*/22aa	Transport proteins for PSM peptides			
			PmtA/298 aa	PmtB/226 aa	PmtC/240 aa	PmtD/246 aa
NCTC8325	WP_001549197.1	no	WP_000991302.1	WP_000645727.1	WP_000763048.1	WP_001221651.1
Newman	WP_001549197.1	no	WP_000991302.1	WP_000645727.1	WP_000763048.1	WP_001221651.1
Col	WP_001549197.1	no	WP_000991302.1	WP_000645727.1	WP_000763048.1	WP_001221651.1
FPR3757	WP_001549197.1	no	WP_000991302.1	WP_000645727.1	WP_000763048.1	WP_001221651.1
N315	WP_001549197.1	WP_014532405.1	WP_000991306.1	WP_000645727.1	WP_000763043.1	WP_001221651.1
MW2	WP_001823225.1	no	n/d	n/d	WP_000763048.1	WP_001221657.1
MRSA252	CAG41103.1 (45aa)	WP_014532405.1	n/d	n/d	WP_000763048.1	n/d
T0131	WP_001549197.1	WP_014532405.1	WP_000991302.1	WP_000645727.1	WP_000763048.1	WP_001221651.1
55/2053	WP_001549197.1	no	n/d	n/d	WP_000763048.1	n/d
TW20	WP_001549197.1	WP_014532405.1	WP_000991302.1	WP_000645727.1	WP_000763048.1	WP_001221651.1
Z172	WP_001549197.1	WP_014532405.1	WP_000991302.1	WP_000645727.1	WP_000763048.1	WP_001221651.1
V521	n/d	n/d	n/d	n/d	n/d	n/d
Bmb9393	WP_001549197.1	WP_014532405.1	WP_000991302.1	WP_000645727.1	WP_000763048.1	WP_001221651.1
Be62	n/d	n/d	n/d	n/d	n/d	n/d
JKD6008	WP_001549197.1	WP_014532405.1	WP_000991302.1	WP_000645727.1	WP_000763048.1	WP_001221651.1
SA943	RAM45554.1	RAM45233.1	RAM45801.1	RAM45802.1	RAM45803.1	RAM45804.1

Supplementary Table S8. The Agr locus proteins amino acid sequence.

Strain	AgrA		AgrB				AgrD			
	Protein accession number	Num-ber of amino acids (aa)	Protein accession number	Num-ber of amino acids (aa)	Query Cover (%)/ Per. Ident (%)	Num-ber of amino acids substitu-tions	Protein accession number	Num-ber of amino acids (aa)	Query Cover (%)/ Per. Ident (%)	Num-ber of amino acids substi-tutions (aa)
NCTC 8325	WP_000688492.1	238	WP_001105707.1	189	100/100	no	WP_001093929.1	46	100/100	no
Newman	WP_000688492.1	238	WP_001105707.1	189	100/100	no	WP_001093929.1	46	100/100	no
Col[1]	WP_000688492.1	238	WP_001105707.1	189	100/100	no	WP_001093929.1	46	100/100	no
FPR3757	WP_000688492.1	238	WP_001105707.1	189	100/100	no	WP_001093929.1	46	100/100	no
N315	WP_000688492.1	238	WP_001105696.1	187	**100/63.43**	**74**	WP_001094921.1	47	**97/47.83**	**25**
MW2	WP_000688492.1	238	WP_001105705.1	187	**100/79.14**	**39**	WP_001093929.1	46	100/100	no
55/2053	WP_000688492.1	238	WP_001105709.1	187	**100/78.07**	**41**	WP_000735197.1	46	**100/52.17**	**22**
MRSA252	WP_00688492.1	238	WP_001105709.1	187	**100/78.07**	**41**	WP_000735197.1	46	100/100	no
T0131	WP_000688492.1	238	WP_001105707.1	189	100/100	no	WP_001093929.1	46	100/100	no
TW20	WP_000688492.1	238	WP_001105707.1	189	100/100	no	WP_001093929.1	46	100/100	no
JKD6008	WP_000688492.1	238	WP_001105707.1	189	100/100	no	WP_001093929.1	46	100/100	no
Z172	WP_000688492.1	238	WP_001105707.1	189	100/100	no	WP_001093929.1	46	100/100	no
V521	AND36710.1	259[2]	WP_001105707.1	189	100/100	no	AND36708.1	46	100/100	no
Bmb9393	WP_000688492.1	238	AGP28987.1	207	**91/99.47[3]**	**1**	WP_001093929.1	46	100/100	no
Be62	WP_000688492.1	238	ALY23941.1	207	**91/99.47[3]**	**1**	WP_001093929.1	46	100/100	no
SA943	RAM 45557.1	238	RAM 45555.1	189	100/100	no	RAM45556.1	46	100/100	no

Supplementary Table S8. The Agr locus proteins amino acid sequence (continued).

Strain	AgrC			
	Protein accession number	Number of amino acids (aa)	Query Cover (%) / Per.Ident (%)	Substitutions/ deletions/ incisions (aa)
NCTC8325	YP_500745.1	**414**	100/**99.76**	***P247T;*** deletion 1-16
Newman	WP_001554031.1	430	100/**99.77**	***P247T***
Col[1]	WP_010956571.1	430	100/100	no
FPR3757	WP_001549202.1	430	100/ **99.53**	***P247T***; ***I309F***
N315	BAB43125.1	**371**	**98/73.35**	Region 69-204: 98 substitutions; deletions: 1-66;427-430; incision: 1-7
MW2	WP_000387809.1	430	100/**76.51**	Region 1-204: 91 substitutions;<br>further: **P247T**; ***P258T; I280L; I297L; S320T; S321R; 345 T/S***
55/2053	WP_000387809.1	430	100/**76.51**	Регион 1-204: 91 substitutions;<br>further: **P247T**; ***P258T; I280L; I297L; S320T; S321R; T345S***
MRSA252	WP_000387814.1	430	100/**76.51**	Region 1-204: 92 substitutions;<br>further: ***247 P/T; 258 P/T; I280L; I297L; S320T; S321R; 345T/S***
T0131	AEB89137.1	**233**	100/**99.1**	Deletions 1-207, substitutions: ***247 P /T; 311 I/T***
TW20	CBI49894.1<br>WP_014532426.1	430	100/**99.53**	***S6R***; **P247T**
JKD6008	WP_001554031.1	430	100/**99.77**	***P247T;***
Z172	WP_014532426.1	430	100/**99.53**	***S6R***; ***P247T;***
V521	WP_014532426.1	430	100/**99.53**	***6 S/R***; ***P247T;***
Bmb9393	WP_001554031.1	430	100/**99.77**	***P247T;***
Be62	WP_001554031.1	430	100/**99**.77	***P247T;***
SA943	RAM45580.1	**414**	100/**99.52**	***P247T; I311T; A343T;*** deletion 1-16

Note: [1] - the analysis was carried out by align to the reference sequence of the protein in strain Col; [2] - the first 237 amino acids are identical, the C-terminal end contains an additional 22 amino acids; [3]- Insertion 1-18aa and substitution ***M1L*** were identified.

Supplementary Table S9. *Staphylococcus aureus* SarA locus proteins amino acid sequence: localization of amino acid substitutions and deletions.

Strain	Sar A			Sar R			Sar S (SarH1)				SarT			
	Protein accession number	Aa[1]	Per. Ident (%)	Protein accession number	Aa[1]	Per. Ident (%)	Protein accession number	Aa[1]	Per. Ident (%)	Aa sub-stitu-tions	Protein accession number	Aa[1]	Per.Ident (%)	Aa sub-stitu-tions
NCTC 8325	WP_001018677.1	124	100	WP_000036076.1	115	100	WP_000876758.1	250	100	no	SQF74841.1	118	100	no
Newman	WP_001018677.1	124	100	WP_000036076.1	115	100	WP_000876758.1	250	100	no	WP_000998869.1	118	100	no
Col	WP_001018677.1	124	100	WP_000036076.1	115	100	AAW38739.1	250	99.20	**D221N D243N**	WP_000998869.1	118	100	no
FPR3757	WP_001018677.1	124	100	WP_000036076.1	115	100	WP_000876758.1	250	100	no	WP_000998869.1	118	100	no
N315	BAB41805.1	124	100	BAB43387.1	115	100	BAB41327.1	250	99.20	**D221N D243N**	BAB43589.1	119	99,15	**D75E**
MW2	WP_001018677.1	124	100	WP_000036076.1	115	100	WP_000876758.1	250	100	no	BAB58660.1	119	99,15	**D75E**
55/2053	WP_001018677.1	124	100	WP_000036076.1	115	100	WP_000876758.1	250	100	no	n/d	-	-	-
MRSA252	WP_001018677.1	124	100	WP_000036076.1	115	100	WP_000876758.1	250	100	no	n/d	-	-	-
T0131	WP_001018677.1	124	100	WP_000036076.1	115	100	AEB87224.1	250	100	no	WP_000998869.1	118	100	no
TW20	WP_001018677.1	124	100	WP_000036076.1	115	100	WP_000876758.1	250	100	no	WP_000998869.1	118	100	no
JKD6008	WP_001018677.1	124	100	WP_000036076.1	115	100	WP_000876758.1	250	100	no	WP_000998869.1	118	100	no
Z172	WP_001018677.1	124	100	WP_000036076.1	115	100	WP_000876758.1	250	100	no	WP_000998869.1	118	100	no
Bmb9393	WP_001018677.1	124	100	WP_000036076.1	115	100	WP_000876758.1	250	100	no	WP_000998869.1	118	100	no
V521	n/d			AND37114.1	115	100	AND34701.1	250	100	no	n/d			
Be62	ALY22452.1	124	100	ALY24202.1	115	100	ALY21934.1	250	100	no	ALY24402.1	118	100	no
SA943	RAM46821.1	124	100	RAM46294.1	115	100	RAM47915.1	250	100	no	RAM47202.1	118	100	no

Supplementary Table S9. *Staphylococcus aureus* SarA locus proteins amino acid sequence: localization of amino acid substitutions and deletions (continued).

Strain	Sar X (HTH-type transcriptional regulator SarX)	Aa[1]	Sar Z (MarR family transcriptional regulator)	Aa[1]	Aa sub-stitu-tions	SarV (MarR family transcriptional regulator**)**	Aa[1]	SarU (sarH2) (HTH-type transcriptional regulator SarU)	Aa[1]	Repressor of toxin Rot (MarR family transcriptional regulator)	Aa[1]	Query Cover (%)	Per Ident (%)
NCTC 8325	n/d	-	WP_000289213.1	148 •	no •	WP_000066900.1	116	WP_000386367.1	247	WP_000757543.1	133	100	100
Newman	WP_001090985.1	119	WP_000289213.1	148 •	no •	WP_000066900.1	116	n/d	-	WP_000757543.1	133	100	100
Col	WP_001090985.1	119	WP_000289213.1	148 •	no	WP_000066900.1	116	n/d	-	Q5HF12.1	166[5]	80	90.25
FPR3757	WP_001090985.1	119	WP_000289213.1	148 •	no •	WP_000066900.1	116	WP_000386366.1	247[2]	WP_000757543.1	133	100	100
N315	WP_001090985.1	119	BAB43476.1<br>WP_000289215.1	148* •	**K147R** •	BAB43359.1	116	WP_000386367.1	247	BAB42851.1<br>WP_000757543.1	153[3]<br>133	86<br>100	100<br>100
MW2	WP_001090985.1	119	WP_000289213.1	148	no	WP_000066900.1	116	WP_000386367.1	247	BAB95570.1<br>WP_000757543.1	153[3]<br>133	86<br>100	100<br>100
55/2053	n/d	-	n/d	-	-	WP_000066900.1	116	n/d	-	EEV03715.1	166[4]	80	100
MRSA252	n/d	-	n/d	-	-	WP_000066900.1	116	n/d	-	Q6GFT9.1<br>WP_000757543.1	166[4]<br>133	80<br>100	100<br>100
T0131	WP_001090985.1	119	AEB89486.1	148	no	WP_000066900.1	116	WP_000386367.1	247	AEB88851.1<br>WP_000757543.1	133	100	100
TW20	WP_001090985.1	119	WP_000289213.1	148	no	WP_000066900.1	116	WP_000386367.1	247	WP_000757543.1	133	100	100
JKD6008	WP_001090985.1	119	WP_000289213.1	148	no	WP_000066900.1	116	WP_000386367.1	247	WP_000757543.1	133	100	100
Z172	WP_001090985.1	119	WP_000289213.1	148	no	WP_000066900.1	116	WP_000386367.1	247	WP_000757543.1	133	100	100
Bmb9393	WP_001090985.1	119	WP_000289213.1	148	no	WP_000066900.1	116	WP_000386367.1	247	WP_000757543.1	133	100	100
V521	AND37581.1	119	n/d	-	-	AND37084.1	116	AND37117.1	247	n/d	-	-	-
Be62	ALY22503.1	119	ALY24649.1	148	no	ALY24173.1	116	ALY24403.1	247	n/d	-	-	-
SA943	RAM47004.1	119	?	-	-	RAM46266.1	116	RAM46810.1	247	RAM45395.1	133	100	100

Note: 1- Aa[1-] number of amino acids; [2]- identities - 99,6; aa substitution **L211H**; [3] – BAB42851.1 and BAB95570.1 are identical and have 20 amino acids additionally at the N-terminus, the remaining 133 amino acids are common with WP_000757543.1; [4] Q6GFT9.1 and EEV03715.1 are identical and have 30 amino acids additionally at the N-terminus, the remaining 133 amino acids are common with WP_000757543.1; Q5HF12.1 is more different, has 30 amino acids additionally at the N-terminus and amino acid substitution **S51F.** The localization of this substitution is indicated when aligned with the protein WP_000757543.1 in reference genome NCTC 8325
